# Genome-wide identification of the *Capsicum* bHLH transcription factor family: discovery of a candidate regulator involved in the regulation of species-specific bioactive metabolites

**DOI:** 10.1186/s12870-021-03004-7

**Published:** 2021-06-07

**Authors:** Renjian Liu, Jiali Song, Shaoqun Liu, Changming Chen, Shuanglin Zhang, Juntao Wang, Yanhui Xiao, Bihao Cao, Jianjun Lei, Zhangsheng Zhu

**Affiliations:** 1 Key Laboratory of Biology and Genetic Improvement of Horticultural Crops (South China), College of Horticulture, Ministry of Agriculture and Rural Affairs, South China Agricultural University, Guangzhou, 510642 Guangdong China; 2 Lingnan Guangdong Laboratory of Modern Agriculture, Guangzhou, 510642 China; 3 Henry Fok College of Biology and Agriculture, Shaoguan University, Shaoguan, 512005 China; 4 Department of Biology, Peking University-Southern University of Science and Technology Joint Institute of Plant and Food Sciences, Southern University of Science and Technology, Shenzhen, 518055 China

**Keywords:** BHLH, Peppers, Carotenoids, Capsaicinoids, Temperature, Yeast two-hybrid assays

## Abstract

**Background:**

The basic helix–loop–helix (bHLH) transcription factors (TFs) serve crucial roles in regulating plant growth and development and typically participate in biological processes by interacting with other TFs. Capsorubin and capsaicinoids are found only in *Capsicum*, which has high nutritional and economic value. However, whether bHLH family genes regulate capsorubin and capsaicinoid biosynthesis and participate in these processes by interacting with other TFs remains unknown.

**Results:**

In this study, a total of 107 CabHLHs were identified from the *Capsicum annuum* genome. Phylogenetic tree analysis revealed that these CabHLH proteins were classified into 15 groups by comparing the CabHLH proteins with *Arabidopsis thaliana* bHLH proteins. The analysis showed that the expression profiles of *CabHLH009*, *CabHLH032*, *CabHLH048*, *CabHLH095* and *CabHLH100* found in clusters C1, C2, and C3 were similar to the profile of carotenoid biosynthesis in pericarp, including zeaxanthin, lutein and capsorubin, whereas the expression profiles of *CabHLH007*, *CabHLH009*, *CabHLH026, CabHLH063* and *CabHLH086* found in clusters L5, L6 and L9 were consistent with the profile of capsaicinoid accumulation in the placenta. Moreover, *CabHLH007*, *CabHLH009*, *CabHLH026* and *CabHLH086* also might be involved in temperature-mediated capsaicinoid biosynthesis. Yeast two-hybrid (Y2H) assays demonstrated that *CabHLH007*, *CabHLH009*, *CabHLH026, CabHLH063* and *CabHLH086* could interact with MYB31, a master regulator of capsaicinoid biosynthesis.

**Conclusions:**

The comprehensive and systematic analysis of CabHLH TFs provides useful information that contributes to further investigation of CabHLHs in carotenoid and capsaicinoid biosynthesis.

**Supplementary Information:**

The online version contains supplementary material available at 10.1186/s12870-021-03004-7.

## Background

Peppers (*Capsicum* spp*.*), which include sweet and hot chili varieties, are important economic crops in the world [1]. Five domesticated *Capsicum* species, namely, *Capsicum annuum*, *Capsicum baccatum*, *Capsicum chinense*, *Capsicum frutescens* and *Capsicum pubescens*, corresponding to a total of 38.1 million tons of hot peppers, are consumed by three-quarters of the world’s population (FAO, see www.fao.org) [1]. Capsorubin and capsanthin can be described as carotenoids and are exclusively biosynthesized in peppers. Peppers are beneficial for preventing various diseases, such as eye diseases, cardiovascular diseases and certain cancers [2–4], and provide excellent natural colourants [5]. The accumulation of carotenoid pigments results in colour variations in the ripe pepper fruit. The carotenoids biosynthetic pathway is currently known (Figure S1A) [6, 7]. Capsorubin or capsanthin is transformed by geranylgeranyl pyrophosphate (GGPP) in a series of enzymatic reactions with phytoene synthase (PSY), phytoene desaturase (PDS), lycopene β-cyclase (LCYB), etc. [6, 7]. Moreover, capsaicinoids, the alkaloids specific to *Capsicum*, are responsible for pungency. Capsaicinoids are produced in the fruit placenta of hot peppers [8]. Capsaicinoids mainly contain capsaicin, dihydrocapsaicin and several analogues [9, 10]. Capsaicin and dihydrocapsaicin comprise approximately 91% of the total capsaicinoid content in *Capsicum* species [11]. Numerous studies have demonstrated that capsaicinoids are produced after the condensation of phenylalanine and in fatty acid chain biosynthetic pathways (Figure S1B) [1, 12, 13].

The bHLH family is one of the largest transcription factors (TFs) in plants and is important for plant growth and development [14, 15]. bHLH proteins were originally found in eukaryotic species [14] and contain a basic and a helix–loop–helix (HLH) region. The two regions possess DNA-binding and protein-interacting abilities [16]. The basic region contains 13–17 basic amino acids in the N-terminal domain and provides a DNA-binding region to bind to consensus hexanucleotide E-box (CANNTG) [17, 18], whereas the HLH region includes approximately 40 amino acids in the C-terminal domain with two alpha helixes separated by a loop of variable length [16, 19]. Furthermore, the HLH region promotes the interaction with other bHLH proteins and the formation of homodimers and heterodimeric complexes [18]. Except for the two conserved regions, the bHLH protein sequences are dissimilar, revealing protein evolution [20].

The bHLH TFs have been identified in the majority of plants, including *Arabidopsis thaliana*, rice, and potato [15, 21–30]. WRKY, NAC and APETALA2/ETHYLENE RESPONSE FACTOR (AP2/ERF) TF families have been analysed systematically in peppers [31–34]. The bHLH TFs are closely related to the primary and specialized metabolites of the plant. PHYTOCHROME INTERACTING FACTOR 3 (PIF3), a bHLH TF, positively regulates anthocyanin biosynthesis by activating the transcription of anthocyanin biosynthetic genes in *Arabidopsis thaliana* [35]. SlAN1 and SlPIF1a regulate anthocyanin and carotenoid biosynthesis in tomato, whereas SlPRE2 negatively regulates pigment accumulation in fruit [36–38]. The bHLH proteins typically regulate secondary metabolite biosynthesis by interacting with TFs such as MYB, WD40-repeat (WDR), and ETHYLENE RESPONSIVE FACTOR (ERF) [39–43]. bHLH TFs physically interact with MYB proteins and facilitate their regulation of anthocyanin biosynthesis in plants. PacMYBA physically interacts with several anthocyanin-related bHLH TFs controlling anthocyanin biosynthesis in sweet cherry by activating the promoters of *PacDFR*, *PacANS* and *PacUFGT* [39]. AtTT8 interacts with MYB proteins PRODUCTION OF ANTHOCYANIN PIGMENT1/PRODUCTION OF ANTHOCYANIN PIGMENT (PAP1/PAP) and TRANSPARENT TESTA2 (TT2) to regulate anthocyanin and proanthocyanidin biosynthesis in *Arabidopsis thaliana* [44, 45]. Rosea1 (ROS1, an MYB type) and Delila (DEL, a bHLH type) collectively regulate anthocyanin biosynthesis in tomato [46]. In peppers, CaMYC combined with CaMYB and CaWD40 regulates anthocyanin biosynthesis through the regulation of the transcription of a synthetic gene [40]. The physical interaction and regulatory synergy between MYB and bHLH TFs are mediated by the R3 domain in MYB proteins and the N-terminal region in bHLH proteins [45, 47–49]. Additionally, bHLH-mediated ERF TFs regulate synthesis of alkaloids, such as terpenoid indoles alkaloids [42], glycoside alkaloids [41], and nicotine [43]. Capsorubin and capsaicinoids are strictly biosynthesized in *Capsicum*, which possesses high economic and nutritional value. Our previous studies confirmed that MYB31, MYB108 and MYB48 were involved in capsaicinoid biosynthesis [1, 12, 50, 51]. Specifically, natural variations of the master regulator MYB31 promoter can affect capsaicinoid contents among different *Capsicum* species [1, 50]. However, whether bHLH TFs regulate capsorubin and capsaicinoid biosynthesis in peppers and orchestrate the regulation of capsaicinoid biosynthesis by interacting with MYB or other TFs needs to be addressed.

In this study, bHLH family genes were identified in the *Capsicum annuum* genome. A reference genome of *C. annuum* was sequenced (2n = 2x = 24), and its genome size was estimated to be 3.48 Gb by 19-mer analysis [52]. Characteristic analysis of bHLH family members was systematically performed for better understanding the potential biological function of candidate bHLH TFs in regulating species-specific metabolite biosynthesis in peppers. Capsaicinoids biosynthesis is affected by environmental factors, such as water, temperature and light [53]. Higher temperatures are beneficial to the accumulation of total capsaicinoids [53]. bHLH TFs respond to temperature in *Camellia sinensis* [26] and *Arabidopsis thaliana* [54]. Thus, the candidate bHLH TFs were also studied in response to different temperatures. Moreover, the interaction between bHLH TFs and MYB31 was analysed by Y2H. This study provides candidate bHLH TFs in carotenoid and capsaicinoid biosynthetic pathways in peppers.

## Results

### Identification and chromosomal localizations of *bHLHs* in pepper

After excluding redundant sequences, a total of 107 bHLH proteins were identified from the *C. annuum* genome using the Hidden Markov Model (HMM) profile of the HLH domain (PF00010). As shown in Table S1, all identified CabHLH proteins encoded 190–940 amino residues. The molecular weight (Mw) of these proteins ranged from 21.61 kDa to 106.70 kDa, and the theoretical pI ranged from 4.60 to 9.91.

The 107 *CabHLHs* were renamed as *CabHLH001* to *CabHLH107* and mapped to the pepper chromosomes based on the chromosomal positions (Figure S2). They were distributed on 12 chromosomes. Chromosome 01 contained the largest number of *CabHLH* members (18). Chromosomes 05 and 09 included three *CabHLH* members, separately. However, the genes ranging from *CabHLH092* to *CabHLH107* were not located on any chromosomes. Genes located in some scaffolds were not assembled to chromosomes due to sequencing and assembly technology limitations.

### Conserved amino acid residues in the bHLH domain

**Fig. 1:**
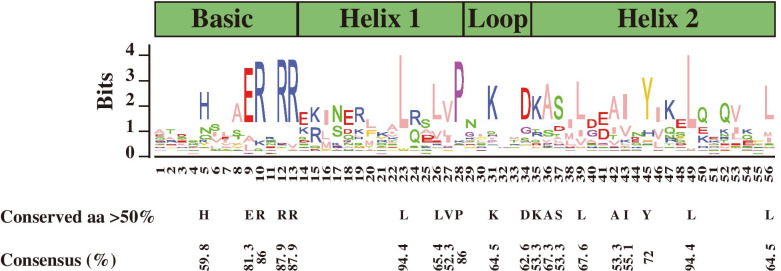
Conserved amino acid analysis of bHLH domains in peppers. The height of the amino acid indicates the frequency observed in all the identified bHLH proteins. The black letters represented the highly conserved amino acids with a consensus ratio greater than 50%

The amino acid sequences of the bHLH domain were used to perform multiple alignment analyses (Figure S3). The results indicated that bHLH family proteins possessed a conserved bHLH domain, which contained the basic, first helix, loop and second helix regions. As shown in Fig. 1, 20 amino acid residues were conserved with a consensus ratio greater than 50%, and six amino acid residues were conserved with a consensus ratio greater than 75% among the conserved bHLH domains. Five residues (His-5, Glu-9, Arg-10, Arg-12 and Arg-13) were conserved in the basic region. Four residues (Leu-23, Leu-26, Val-27 and Pro-28) were conserved in the first helix region. Lys-31 and Asp-34 were conserved in the loop region, and nine residues (Lys-35, Ala-36, Ser-37, Leu-39, Ala-42, Ile-43, Tyr-45, leu-49 and leu-56) were conserved in the second helix region. The residues Leu-23 and Leu-49 were extremely conserved among the 107 bHLH proteins in pepper.

### Phylogenetic analysis of the bHLH family proteins

**Fig. 2:**
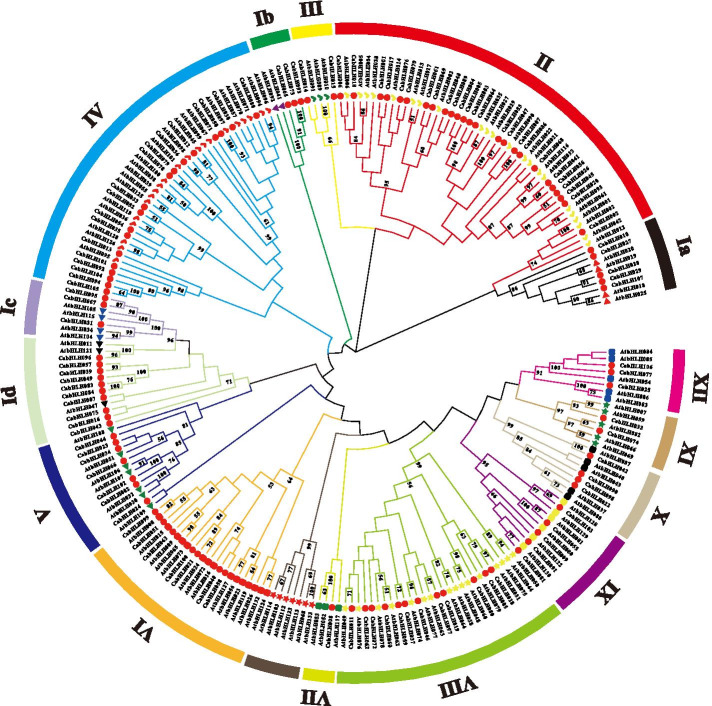
The phylogenetic tree of the bHLH family members in *Capsicum* and *Arabidopsis thaliana*. The differently coloured branches indicated different subgroups. Red circles represented CabHLH proteins. Different colours and shapes represented different groups of AtbHLH proteins identified in a previous study [21]. The brown branch indicated the absence of CabHLH proteins

To classify the CabHLH proteins, a phylogenetic tree that contained all of the identified bHLH protein sequences in peppers and those in *Arabidopsis thaliana* was constructed with the neighbour-joining method (Fig. 2). According to the classification of AtbHLHs in a previous study [21], the CabHLH proteins were divided into 15 subfamilies and named groups I to XII. Group II contained the largest numbers of CabHLHs (25) and AtbHLHs (13), whereas group VII contained only one CabHLH and three AtbHLH proteins. The different number of CabHLHs and AtbHLHs proteins in the same group might arise from unequal duplication of the bHLH family during the plant's evolutionary process.

Members of the same group might possess similar biological functions. To preliminarily speculate on the biological functions of CabHLHs, another neighbour-joining phylogenetic tree was constructed using all bHLH proteins in *Arabidopsis thaliana*, tomato, rice and pepper (Figure S4). The functional characteristics of AtbHLHs and SlbHLHs have been reported and summarized in the literature, and these functional characteristics were used to evaluate the potential function of CabHLH in the same group (Table S2). SlPIF1a included in group VI could regulate carotenoid biosynthesis by a light-dependent mechanism in tomato [38]. SlPIF1a mapped to *CabHLH051* in peppers and was also classified into group VI. These results indicated that the members of group VI might be involved in carotenoid biosynthesis.

### Analysis of conserved motifs in CabHLH proteins

**Fig. 3:**
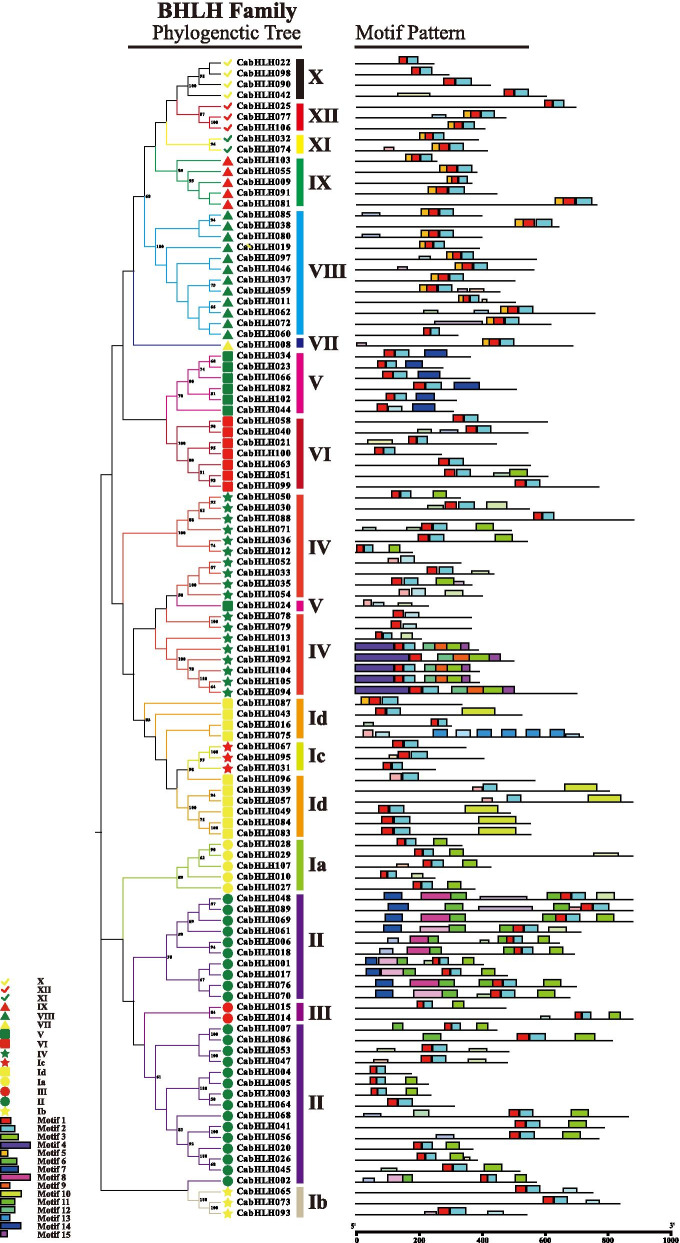
Phylogenetic tree and conserved motif distributions of CabHLH proteins. The neighbour-joining phylogenetic tree was constructed with MEGA-X. Different coloured boxes represent different motifs. Box length represents motif length. Differently coloured shapes indicate different groups in the phylogenetic tree of CabHLHs

To investigate the structural features of CabHLH proteins, the conserved amino acid motifs were analysed and identified using the Multiple EM for Motif Elicitation (MEME) Suite. A total of 15 conserved motifs containing 21–100 residues were found in motifs 1 to 15. The motifs information is provided in Table S3. Motifs 1 and 2 were located in the bHLH domain region, which appeared in all proteins. Motifs 3 to 15 were distributed outside the bHLH domain region. Motifs 6, 7, 8 and 11 were primarily restricted to group II. Motifs 9, 12 and 15 were specifically identified within group IV. Motifs 10 and 13 were found in group Id, and motif 14 was observed in group V. Generally, most of the same group’s proteins possessed common motifs in terms of alignment and position (Fig. 3).

### Expression profiles of *CabHLHs* at different developmental stages in pericarp and placenta

**Fig. 4:**
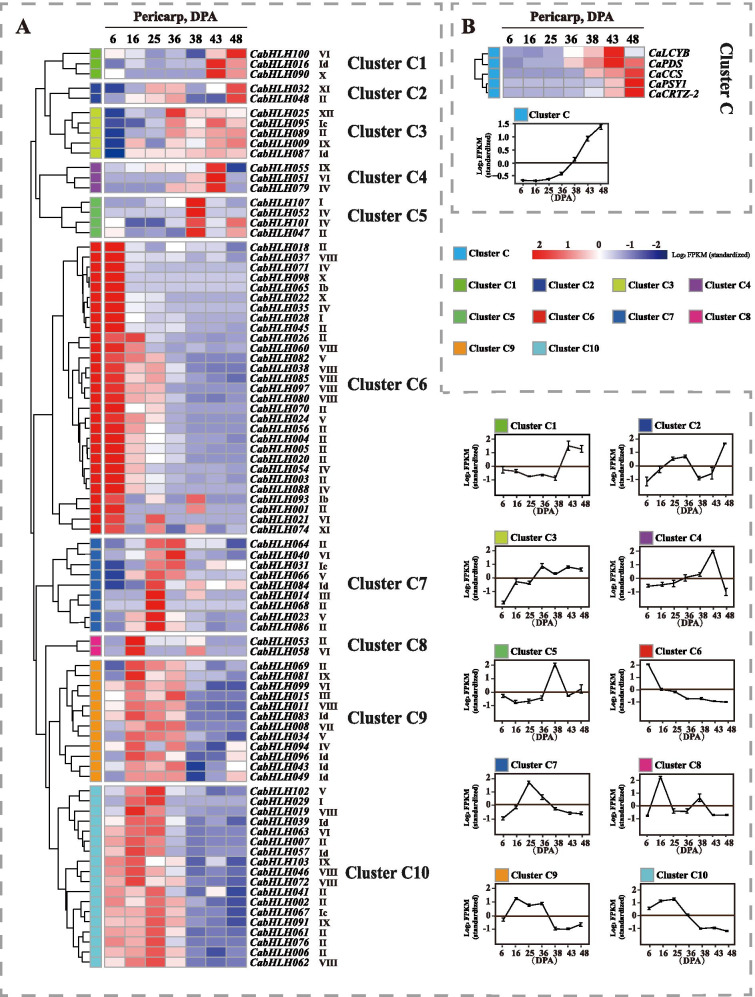
Expression profiles of the genes in the pericarp at different developmental stages. **a**. Expression profiles of *CabHLH* genes at different developmental stages of pericarp. **b**. Expression profiles of the capsorubin biosynthetic genes at different developmental stages of pericarp. The name of each gene and the short name of the phylogenetic group were included to the right of the heat map. Log2 values of fragments per kilobase of exon per million fragments mapped (FPKM) were used to construct the heat map based on the hierarchical clustering analysis. Line charts were generated using the mean value for the whole cluster. The letter “C” in cluster C indicates the pericarp

**Fig. 5:**
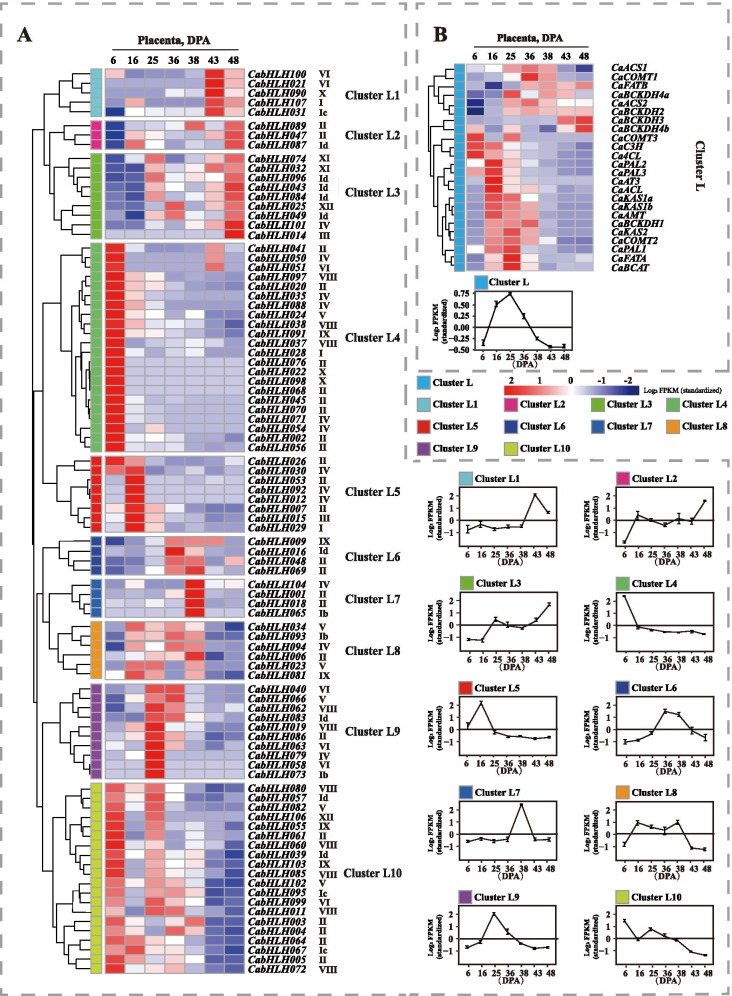
Expression profiles of the genes in the placenta at different developmental stages. **a**. Expression profiles of *CabHLH* genes at different developmental stages of placenta. **b**. Expression profiles of capsaicinoid biosynthetic genes at different developmental stages of placenta. The name of each gene and the short name of the phylogenetic group appear to the right of the heat map. Log2 values of FPKM were used to construct the heat map with hierarchical clustering analysis. Line charts were prepared using the mean value for the whole cluster. The letter “L” in cluster L indicates the placenta

Capsorubin and capsaicinoids are found in pepper fruit and closely associated with the biosynthetic genes transcriptional level during developmental stages. To obtain further insight into the potential functions of CabHLHs during capsorubin and capsaicinoid biosynthesis, the expression profiles of *CabHLHs* in different developmental pericarp and placenta were investigated. RNA-Seq raw data were obtained from Kim et al. [52] and included 6, 16, 25, 36, 38, 43 and 48 days post-anthesis (DPA) stages (Figs. 4 and 5). All the raw reads were spliced and remapped to version 2.0 of the *C. annuum* genome.

As shown in Fig. 4b, the expression levels of capsorubin biosynthetic genes gradually increased at 36 DPA, which was consistent with the accumulation profile of capsorubin in pericarp tissue. A total of 20 expressed *CabHLH* genes were not detectable. These genes were probably transcribed at a low level in different developmental pericarp stage. According to the similarity of the expression profiles, all *CabHLH* expression profiles in different developmental pericarp stages were hierarchically clustered and classified into 10 clusters (Fig. 4a). The expression profiles of *CabHLHs* in clusters C1 to C4 maintained good agreement with the expression profiles of capsorubin biosynthetic genes. The members of clusters C1 to C4 might be associated with capsorubin biosynthesis.

Capsaicinoids were abundantly produced from 13 to 25 DPA in the placenta's developmental stages, and the expression levels of capsaicinoid biosynthetic genes were high during this stage (Fig. 5b). Based on the similarity of the expression profiles, the expression of all the CabHLHs in different developmental placenta stages were hierarchically clustered into 10 clusters (Fig. 5a). A total of 16 genes were expressed at a low level that could not be detected. The expression profiles of *CabHLHs* in clusters L5, L6, L8 and L9 were similar to the expression profiles of capsaicinoid biosynthetic genes. Therefore, these results indicated that the members of clusters L5, L6, L8 and L9 might be associated with capsaicinoids biosynthesis.

Additionally, capsorubin and capsaicinoids are produced mainly in the pericarp and placental tissues of peppers. To confirm whether *CabHLHs* were specifically expressed in pericarp and placental tissues, the expression profiles of all identified CabHLHs in different tissues, including the leaf, root, stem, pericarp and placenta, were investigated. However, the RNA-Seq raw data uploaded by Kim et al. did not contain leaf, root or stem tissues from peppers [52]. The RPKM values of these tissues, which were mapped to version 1.5 of the *C. annuum* genome, were directly published online. The heat map indicated that *CabHLHs* were not specifically expressed in certain tissues (Figure S5). Presumably, these TFs orchestrated functions in addition to regulating capsorubin and capsaicinoid biosynthesis.

### Validation of candidate bHLH genes involved in capsorubin and capsaicinoids biosynthesis

**Fig. 6:**
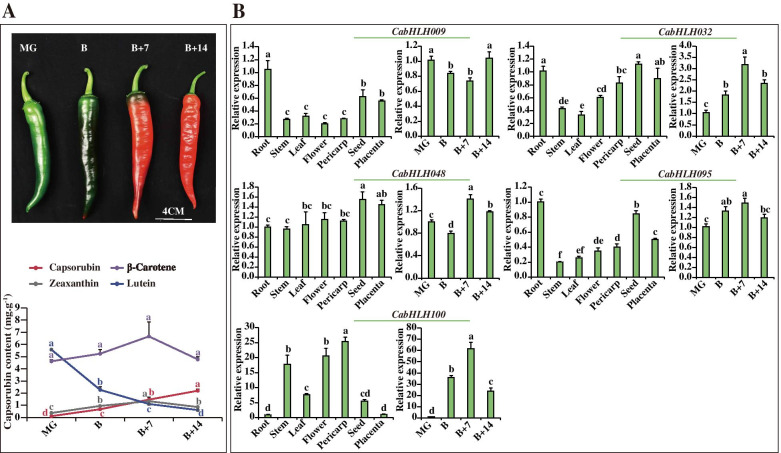
Expression profiles of five *CabHLHs* in different tissues and developmental stages of pericarp. **a**. Phenotypes and the contents of β-carotene, zeaxanthin. lutein and capsorubin at four developmental pericarp stages, namely, the mature green (MG), breaker (B), breaker plus 7 days (B + 7) and breaker plus 14 days (B + 14) stages [34]. **b**. The expression of five *CabHLHs* in different tissues (stem, root, seed, flower, leaf, pericarp (B) and placenta (16 DPA)) and four different pericarp stages. Different letters in the figures indicate significantly different values (*P* < 0.05, Tukey’s test). Three biological replicates of all tissues and developmental pericarp stages were performed with three technical replicates of each biological replicate

To further verify the expression profiles of *CabHLHs* in the pericarp and placenta, ten *CabHLHs* from candidate clusters that might be related to capsorubin and capsaicinoid biosynthesis were selected for qRT-PCR analysis. The selected genes were relatively highly expressed in different developmental pericarp or placental tissues. As shown in Fig. 6a, the contents of zeaxanthin and capsorubin increased from the MG stage in pericarp tissue, whereas the lutein content from the branch of the non-synthetic capsorubin decreased. No significant difference in β-carotene content was noted in different developmental pericarp tissues. *CabHLH032*, *CabHLH048*, *CabHLH095* and *CabHLH100* expression was consistent with the accumulation profile of carotenoids (zeaxanthin and capsorubin) in pericarp, whereas *CabHLH009* expression was similar to the accumulation profile of lutein in pericarp. However, these genes were also highly expressed in other tissues (roots, flowers, stems, placentas, leaves and seeds) (Fig. 6b). Thus, it was likely that the members of clusters C1, C2 and C3 were associated with capsorubin biosynthesis but also possessed other specific functions in other tissues.

**Fig. 7:**
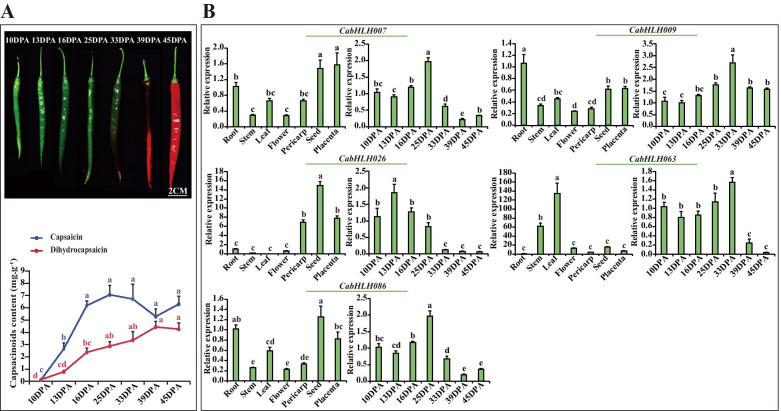
Expression of five *CabHLHs* in different tissues and different developmental placental stages. **a**. Phenotypes (up) and the content of capsaicin and dihydrocapsaicin (down) at seven developmental placental stages, including 10, 13, 16, 25, 33, 39 and 45 DPA stages [34]. **b**. The expression of five *CabHLHs* in different tissues (root, stem, leaf, flower, seed, placenta (16 DPA) and pericarp (16 DPA)) and seven different developmental placenta stages. Different lower-case letters in the figures indicated significantly different values (*P* < 0.05, Tukey’s test). Three biological replicates of all tissues and developmental placental stages were performed with three technical replicates of each biological replicate

The capsaicin and dihydrocapsaicin were initially produced at 10 DPA, peaked at 25 DPA in placental tissue, and then gradually decreased (Fig. 7a). *CabHLH007*, *CabHLH009*, *CabHLH026, CabHLH063* and *CabHLH086* expression resembled the accumulation profile of capsaicin in different developmental placenta stages. A high expression level of *CabHLH026* was observed in pericarp, seed and placental tissues, and high expression of *CabHLH063* was evident in stem and leaf. *CabHLH007*, *CabHLH009* and *CabHLH086* were highly expressed in certain tissues (Fig. 7b). Therefore, *CabHLH007*, *CabHLH009*, *CabHLH026, CabHLH063* and *CabHLH086* in clusters L5, L6 and L9 might be associated with capsaicinoid biosynthesis.

### The expression of candidate *CabHLHs* associated with capsaicinoid biosynthesis in response to different temperatures

**Fig. 8:**
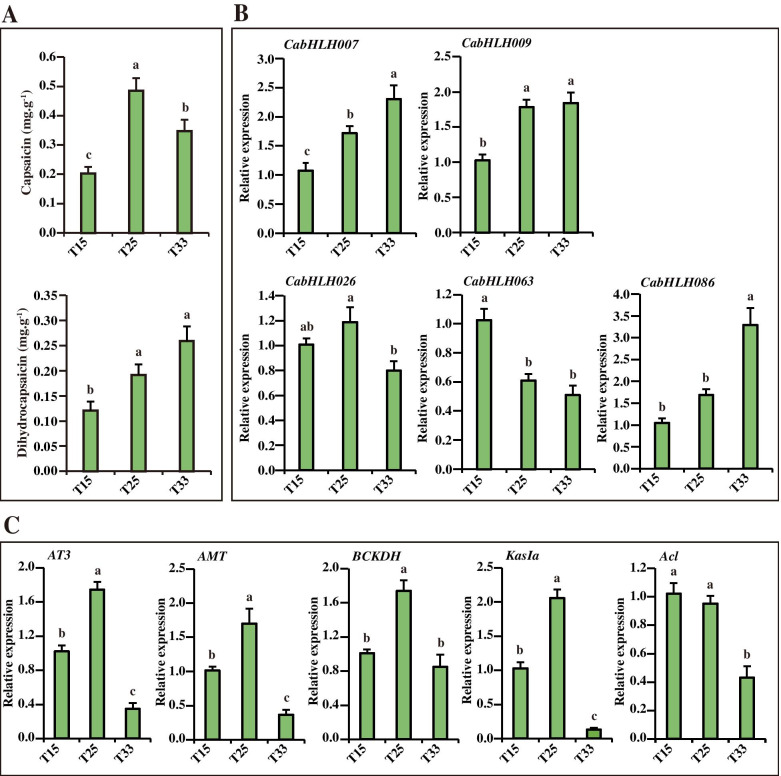
The capsaicinoid content (capsaicin and dihydrocapsaicin) and gene expression in response to different temperatures. **a**. The content of capsaicin and dihydrocapsaicin in response to different temperatures [34]. **b**. The expression of five *CabHLH*s in response to different temperatures. **c**. The expression of capsaicinoid biosynthetic genes in response to different temperatures. Different lower-case letters in the figures indicated significantly different values (*P* < 0.05, Tukey’s test). Three biological replicates of all developmental placental stages were performed with three technical replicates of each biological replicate. T15 = 15℃, T25 = 25℃, and T33 = 33℃

To obtain a preliminary understanding of whether capsaicinoid biosynthesis is regulated by *CabHLH* genes in response to different temperatures, the expression of five candidate *CabHLHs* and five important capsaicinoid biosynthetic genes at different temperatures was measured. As shown in Fig. 8a, the capsaicin and dihydrocapsaicin content tended to increase with increasing temperature from T15 to T25. The placenta capsaicin content with T25 treatment was significantly increased compared with that in peppers with T33 treatment. *CabHLH007*, *CabHLH009* and *CabHLH086* expression increased with increasing temperatures, which was similar to the accumulation of dihydrocapsaicin (Fig. 8b). *CabHLH026* was highly expressed in T25, which was consistent with the accumulation of capsaicin and the expression of capsaicinoid biosynthetic genes (*AT3*, *AMT*, *BCKDH* and *KasIa*) (Fig. 8b; c). In contrast, *CabHLH063* expression decreased with increasing temperature, which maintained consistency with the expression profile of the capsaicinoid biosynthetic gene *Acl* (Fig. 8b; c). Thus, *CabHLH007*, *CabHLH009* and *CabHLH086* expression was positively associated with an increase in dihydrocapsaicin content and temperature. *CabHLH063* expression was negatively related to the increase in the capsaicin content and temperature, whereas *CabHLH026* expression was positively related to these factors. These candidate genes might be related to capsaicinoid biosynthesis in response to different temperatures by regulating the transcription of capsaicinoid biosynthetic genes.

### The interaction of candidate CabHLHs and MYB31 in yeast and identified bHLH binding sites

bHLH proteins typically bind to E-box binding sites of gene promoter and regulate the transcription. To characterize the potential association of bHLH TFs and the pathway biosynthetic genes, one thousand five hundred base pair nucleotide sequences upstream of the start codon (ATG) from capsorubin and capsaicin biosynthetic genes, including *CCS*, *PSY*, *β-CH*, *β-LCY*, *Acl*, *AMT*, *AT3*, *BACT, BCKDH*, *COMTa*, *FatA* and *KasIa*, were analysed using the PlantCARE database [55]. As shown in Table S7, multiple bHLH DNA binding sites were detected in the promoters of capsorubin and capsaicin biosynthetic genes, except for *CCS*, *β-CH* and *KasIa*.

**Fig. 9:**
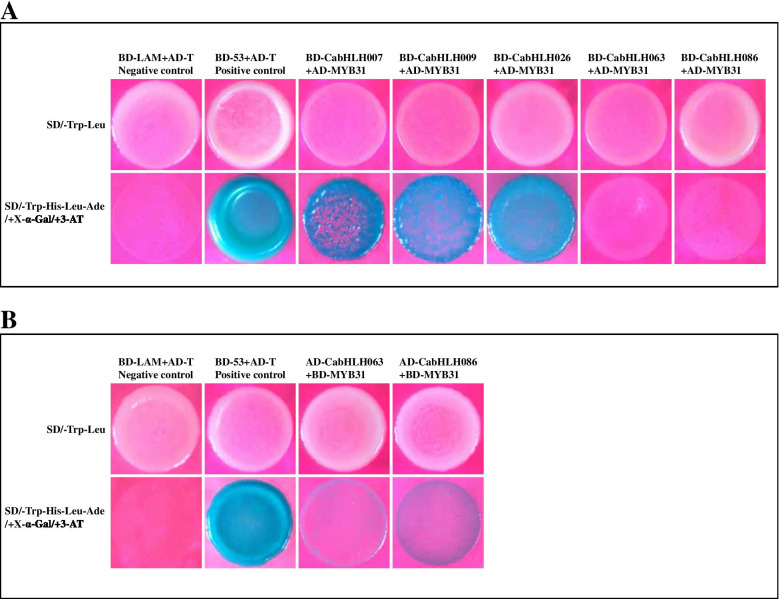
Yeast two-hybrid assays of the protein interactions between candidate CabHLHs and CaMYB31. **a**. Yeast two-hybrid analysis of protein–protein interactions between CabHLHs and CaMYB31. **b**. Reverse yeast two-hybrid analysis of protein–protein interactions between CabHLHs and CaMYB31. AD and BD represented empty pGADT7 and pGBKT7 vectors. SD/-Trp-Leu, the synthetic dextrose (SD) medium lacked tryptophan and leucine; the SD/-Trp-His-Leu-Ade medium lacked tryptophan, histidine, leucine and adenine

The bHLH protein executes function always by interacting with other TFs, such as MYB. We performed Y2H assay to verify the interaction between candidate CabHLHs and CaMYB31. The results indicated that these bHLHs interacted with MYB31 in a gene-dependent manner. CabHLH007, CabHLH009, CabHLH026, CabHLH063 and CabHLH086 could interact with MYB31 in a heterologous system. CabHLH026 strongly interacts with CaMYB31 in yeast, whereas only weak interactions were observed in the group of CabHLH063-CaMYB31 and CabHLH086-CaMYB31 (Fig. 9). Therefore, it was likely that CabHLH could regulate capsaicinoid biosynthesis by interacting with CaMYB31, a master regulator of capsaicinoid biosynthesis.

## Discussion

The bHLH family has emerged as the second-largest TF family in plants [48]. This family has been successfully identified and investigated in many plants, including *Solanum tuberosum L.* (124) [24], *Camellia sinensis (L.) O. Ktze.* (120) [26], *Solanum lycopersicum* (159) [30], *Zea mays* L*.* (208) [28], *Brassica rapa pekinensis* (230) [27], *Arabidopsis thaliana* (147) [15], *Glycine max (*L*)* Merr*.* (319) [56], *Malus pumila* (188) [57] and *Oryza sativa* L*.* (167) [22]. In this study, 107 CabHLH genes were identified in the pepper genome. The conserved bHLH domain consisted of the basic, first helix, loop and second helix regions, which contained approximately 60 amino acids [22]; this structure was also observed in peppers (Figure S3). Twenty amino acid residues were conserved with a consensus ratio greater than 50% (Fig. 1), consistent with a previous study [21]. Glu-9, Arg-12 and Arg-13 were conserved with a consensus ratio greater than 80%, whereas Leu-23 and Leu-49 were extremely conserved among 107 CabHLH proteins (Fig. 1). A previous study had confirmed that Glu-13 and Arg-16/Arg-17 (corresponding to Glu-9 and Arg-12/Arg-13 in this study; Fig. 1) played an important role in DNA binding [58]. Leu-27 and Leu-61 (corresponding to Leu-23 and Leu-49 in this study; Fig. 1) are necessary for dimerization [23]. Glu-13 and Arg-16 (corresponding to Glu-9 and Arg-12 in this study; Fig. 1) recognize the E-box (5’-CANNTG-3’), whereas the His/Lys-9, Glu-13 and Arg-17 (corresponding to His-5, Glu-9 and Arg-13 in this study; Fig. 1) were required for binding G-Box (5’-CACGTG-3’) [19, 59].

According to a previous study [21], the phylogenetic tree was divided into 15 subgroups (Fig. 2), which was similar to the classification of potato [24]. Motifs 1 and 2 located in the bHLH domain were included in all CabHLH proteins (Fig. 3), which was consistent with the observation of MdbHLH proteins [57]. Motifs 1 and 2 containing some conserved amino residues played important roles in DNA binding and protein dimerization [23, 58]. The motifs from 3 to 15 were distributed outside the bHLH domain and randomly arranged in CabHLH proteins (Fig. 3). The results indicated that the members of the same group had the same motif arrangements in most case.

Homologous genes generally possessed a similar function in plant growth and development processes [60]. Some SlbHLHs and AtbHLHs included in groups II and VI were functionally identified (Table S2). MYC2 interacted with MYB TFs to regulate glucosinolate biosynthesis [61] and respond to abscisic acid [62], jasmonic acid [63], and light signalling [64] in *Arabidopsis thaliana*. TRANSPARENT TESTA8 (TT8), GLABRA3 (GL3) and ENHANCER OF GLABRA3 (EGL3) combined with MYB and WDR TFs could regulate anthocyanin biosynthesis and trichome development in *Arabidopsis thaliana* [65–67]. These proteins were in group II (Figure S4). Some SlbHLHs in group II were also involved in anthocyanin biosynthesis [36] and trichome development [68] and responded to cold, osmotic and salt stress [69]. Furthermore, MYC2 interacted with ERF TFs to regulate the biosynthesis of alkaloids such as steroidal glycoalkaloids [41], terpenoid indole alkaloids [42], and nicotine biosynthesis [43]. The homologous genes of MYC2 in pepper were mapped to *CabHLH006* and *CabHLH018*, and all of the proteins were classified into group II (Figure S4). These results indicated that group II might be related to secondary metabolites, development and signal response processes in plants. In addition, PIF3 combined with bZIP TFs could regulate anthocyanin biosynthesis [35]. PIFs served as cellular signalling hubs that function by integrating multiple signals to regulate the transcriptional network during plant growth and development [70]. SlPIF1a was included in group VI and regulated carotenoid biosynthesis in tomato [38]. Homologous genes of these PIFs were mapped to *CabHLH063* and *CabHLH051* in pepper, and the proteins were located in group VI. The CabHLH protein members in groups II and VI probably regulated secondary metabolite biosynthesis by interacting with other TFs.

The expression profiles of *CabHLHs* in clusters C1, C2, C3 and C4 maintained good agreement with the expression of capsorubin biosynthetic genes (Fig. 4). The expression profiles of *CabHLH009*, *CabHLH032*, *CabHLH048*, *CabHLH095* and *CabHLH100* from these clusters aligned with the accumulation profile of carotenoids, including zeaxanthin, lutein and capsorubin, in pericarp (Fig. 6). Therefore, it was likely that the members of these clusters were related to capsorubin biosynthesis. The expression profiles of *CabHLHs* in clusters L5, L6, L8 and L9 were similar to the expression profiles of capsaicinoid biosynthetic genes (Fig. 5). *CabHLH007*, *CabHLH009*, *CabHLH026*, *CabHLH063* and *CabHLH086* expression profiles were consistent with the accumulation profile of capsaicinoids in placenta (Fig. 7). These results indicated that the members of clusters L5, L6 and L9 might regulate capsaicinoid biosynthesis. Moreover, the similar transcript levels of the genes indicated that they might perform similar functions in some cases. CaMYC combined with CaMYB and CaWD40 to regulate anthocyanin biosynthesis [40] and was mapped to *CabHLH0016* in cluster C1 (Fig. 4). Numerous studies have reported that MYC2 typically regulated alkaloid biosynthesis by interacting with ERF TFs [41–43], and ERF TFs were associated with capsaicinoid biosynthesis in *Capsicum* [34, 71]. MYC2 was mapped to two homologous genes CabHLH006 and CabHLH018 that were classified into clusters L8 and L7, respectively. The expression profile of cluster L8 maintained good agreement with the profile of capsaicinoid biosynthesis. SlPIF1a was related to carotenoid biosynthesis in tomato [38] and mapped to *CabHLH051* in the pepper, which was classified into cluster C4. The members of cluster C4 were candidate regulators for carotenoid biosynthesis in this study. The members of clusters C1, C2, C3, C4, L5, L6, L8 and L9 were probably related to carotenoid and capsaicinoid biosynthesis.

Environmental factors are essential for secondary metabolite biosynthesis. Previous studies have demonstrated that bHLH TFs were responsive to a heat response in *Camellia sinensis* and *Arabidopsis thaliana* [26, 72]. PIFs acted as a signalling centre that integrated multiple signals to regulate plant growth and development, including high temperature and light [70]. The expression of CabHLH063, a homologous gene of PIF, and *Acl* decreased with increasing temperature. The expression of *CabHLH026* and four capsaicinoid biosynthetic genes (i.e., *AT3*, *AMT*, *BCKDH* and *KasIa*) peaked in T25, which was similar to capsaicin accumulation (Fig. 8c). These results indicated that the members of clusters L5, L6 and L9 might possess different functions in regulating capsaicinoid biosynthesis in response to different temperatures. In addition, bHLH proteins were typically involved in plant metabolic pathways by interacting with other TFs [39, 41–46]. MYB TFs played a key role in capsaicinoid biosynthesis as evidenced in previous studies [1, 12, 50, 51]. Y2H assays revealed that CabHLH007, CabHLH009, CabHLH026, CabHLH063 and CabHLH086 could interact with MYB31 in a heterologous system. These results implied that CabHLH007, CabHLH009, CabHLH026, CabHLH063 and CabHLH086 might be involved in capsaicinoid biosynthesis by interacting with MYB31.

## Conclusions

A total of 107 CabHLH proteins were identified in peppers. These proteins were divided into 15 groups according to the classification of *Arabidopsis thaliana*. The bHLH conserved domain containing motifs 1 and 2 appeared in all CabHLHs. The expression profiles showed that clusters C1, C2, C3, C4, L5, L6, L8 and L9 were candidates for the regulation of carotenoid and capsaicinoid biosynthesis. *CabHLH009*, *CabHLH032*, *CabHLH048*, *CabHLH095*, *CabHLH100*, *CabHLH007*, *CabHLH009*, *CabHLH026, CabHLH063* and *CabHLH086* were selected from the candidate clusters because they might contribute to carotenoid and capsaicinoid biosynthesis. *CabHLH007*, *CabHLH009*, *CabHLH026, CabHLH063* and *CabHLH086* might be responsive to different temperatures to mediate capsaicinoid biosynthesis. CabHLH007, CabHLH009, CabHLH026, CabHLH063 and CabHLH086 likely control capsaicinoid biosynthesis by interacting with MYB31. However, further studies showing how these candidate bHLHs regulate carotenoid and capsaicinoid biosynthesis are required.

## Methods

### Identification of *bHLH*s in peppers and their chromosomal locations

The bHLH protein sequences were retrieved from *C. annuum* ‘CM334’ genome [73] using the HMM profile of the HLH domain (PF00010) obtained from the PFAM database [74]. Redundant sequences were filtered with HMMER software [75], SMART database [76] and NCBI Conserved Domain Search Service (CD Search) [77]. The ExPASy server [78] was used to predict the full length of amino acid sequences, MW, PI and instability index of the proteins. The corresponding information is provided in Table S1. The chromosomal locations of gene loci were acquired from version 2.0 of the pepper genome. According to the chromosomal positions, all the identified *CabHLH*s were renamed consecutively.

### Multiple alignments and phylogenetic analysis

Multiple sequence alignments were performed using conserved bHLH domain sequences of four plant species, including peppers, rice, tomato and *Arabidopsis thaliana*. The alignments were performed with ClustalX 2.1 with the default parameters. The bHLH protein sequences in *Arabidopsis thaliana* (131) were obtained from The Arabidopsis Information Resource (TAIR) database [79], whereas the bHLH protein sequences in rice (144) and tomato (145) were acquired from Plant TF Database version 4.0 [80]. All of these bHLH protein sequences were renamed and listed in Table S4. The neighbour-joining phylogenetic trees were generated using MEGA X with 1000 bootstrap replications [81] and visualized using EvolView v3 [82].

### Protein motif analysis

Conserved and functional motifs were analysed using the MEME tool [83] with parameters according to Song et al. [34].

### RNA-Seq data and expression profiles of *CabHLH*s

FPKM values of candidate CabHLHs were obtained from the pepper RNA-Seq raw data (GenBank: AYRZ00000000) [84, 85]. Heat maps showing the gene expression profiles in seven different developmental placenta and pericarp tissues (6 DPA to 48 DPA) were drawn by R language.

### Plant materials and temperature treatments

The 59 inbred line (*Capsicum annuum*) authenticated by Professor Jianjun Lei (College of Horticulture, South China Agricultural University) was used for the experiments [1]. The variety rights of this breed belong to our lab. If somebody needs this inbred line, they can acquire from the corresponding authors. All plant materials used in this study were provided by South China Agricultural University. According to institutional, national and international guidelines, these samples do not require specific permissions for research purposes. The seeds were sown using mixture substrate (peat, perlite and coir pith) in the plastic greenhouse under the following environment conditions: 35℃/23℃(day/night); 12 h/12 h (light/dark cycle), and 300–1000 μmol·m^−2^·s^−1^ of photosynthetic photon flux density (PPFD) at 12:00 AM. The sample used for RNA extraction was collected according to the methods of our previous study [34].

Peppers used for temperature treatment experiment were also cultivated using mixture substrate as described above. The temperature treatment and sample collection were performed as previously described [34]. All the samples were performed with three biological replicates and one technical replicate for each biological replicate.

### Quantitative Real-time PCR (qRT-PCR) analysis

The isolation and extraction of total RNA and RNA reverse transcription were performed according to Song et al. [34]. The primers for qPCR were designed using Primer 5.0 (Table S5). Ploy (A)-binding protein (*CA00g52140*) and ubiquitin extension protein (*CA12g20490*) were used as housekeeping genes, given that transcriptome studies revealed that their transcription levels were fairly stable among different tissues and plant different development stages in *Capsicum* [52]. qRT-PCR was performed according to Wang et al. [86]. The relative expression of each *CabHLH* was calculated using the 2^−ΔΔCt^ method [87]. Three technical replicates of each biological replicate and three biological replicates were performed for all samples. The Dunnett’s *t*-test was used to determine the results of significant differences by using SPSS 22.

### Yeast two-hybrid assay

The full-length cDNAs of *CabHLH007*, *CabHLH009*, *CabHLH026, CabHLH063* and *CabHLH086* were cloned into a pMD19-T vector (6013, TaKaRa, China). The amplified full-length fragments of *CabHLH007*, *CabHLH009*, *CabHLH026, CabHLH063* and *CabHLH086* were ligated into the pGADT7 and pGBKT7 vectors using one-step cloning (C112, Vazyme, China), separately. Negative and positive plasmids and plasmids containing different CabHLH were transformed into the AH109 yeast strain (YC1010, Weidi Biotechnology, China). The Y2H assay was performed according to the manufacturer’s instructions (Clontech). Image processing was performed using Adobe Illustrator CS2020. All the primers were listed in Table S6.

### Promoter *cis*-element discovery

The basis region of the bHLH protein could bind to E-box (CANNTG). One thousand five hundred base pair nucleotide sequences upstream of the start codon in the biosynthetic genes of capsorubin (*CCS*, *PSY*, *β-CH*, *β-LCY*) and capsaicin (*Acl*, *AMT*, *AT3*, *BACT, BCKDH*, *CoMTa*, *FatA* and *KasIa*) were retrieved from *C. annuum* genome and applied to E-box discovery. The *cis*-elements were identified by using the PlantCARE database (http://bioinformatics.psb.ugent.be/webtools/plantcare/html/).

## Supplementary Information

**Additional file 1: Figure S1.** Carotenoid (A) and capsaicinoid (B) biosynthetic pathways. **Figure S2.** Chromosomal localizations of *CabHLH*s. **Figure S3.** Multiple alignment analysis of the bHLH domain in pepper bHLH proteins. **Figure S4.** Phylogenetic tree of the bHLH family in *Capsicum annuum*, *Arabidopsis thaliana*, tomato and rice. **Figure S5.** The expression profiles of CabHLH genes in different tissues. **Figure S6.** Diagram of yeast two-hybrid assays experiments

**Additional file 2: Table S1.** The corresponding information of 107 *CabHLH*s. **Table S2.** Functionally characterized partial bHLH proteins from tomato and *Arabidopsis thaliana.* **Table S3.** Features of the CabHLH proteins motifs. **Table S4.** List of the bHLH genes in rice, tomato and *Arabidopsis thaliana.* **Table S5.** List of the primer for real-time quantitative PCR. **Table S6.** List of the primer for yeast two-hybrid assays. **Table S7.** Putative *cis*-elements of capsorubin and capsaicin biosynthetic genes promoters.

## Data Availability

Most data generated or analysed during this study are included in this article and its supplemental files. The sequencing data (GenBank: AYRZ00000000) used and analysed during this study are available in the NCBI database (doi:10.1038/ng.2877).

## Abbreviations

TFs: Transcription factors
Chr: Chromosome
FPKM: Fragments per kilobase of transcript per million mapped reads
bHLH: Basic helix–loop–helix
HLH: Helix–loop–helix
MEME: Multiple EM for Motif Elicitation
HMM: Hidden Markov model
GGPP: Geranylgeranyl pyrophosphate
PSY: Phytoene synthase
PDS: Phytoene desaturase
LCYB: Lycopene β-cyclase
Pal: Phe ammonia-lyase
COMT: Caffeic acid O-methyltransferase
AT3: A putative acyltransferase
CCS: Capsanthin/capsorubin synthase
CS: Capsaicin synthase
ZDS: ζ-Carotene desaturase
CRTISO: Carotenoids isomerase
LCYE: Lycopene ε-cyclase
CrtZ-2: β-Carotene hydroxylase-2
PAL: Phenylalanine ammonia-lyase
C4H: Cinnamate 4-hydroxylase
4CL: 4-Coumaroyl-CoA ligase
HCT: Hydroxycinnamoyl transferase
C3H: p-Coumaroyl shikimate/quinate 3-hydroxylase
COMTa: Caffeoyl-CoA 3-O-methyltransferase
HCHL: Hydroxycinnamoyl-CoA hydratase lyase
AMT: Aminotransferase
BCAT: Branched-chain amino acid aminotransferase
Kas: Ketoacyl-ACP synthase
ACL: Acyl carrier protein
FatA: Acyl-ACP thioesterase
DPA: Days post-anthesis
MG: Mature green stage
B: Breaker
B + 7: Breaker plus 7 days
B + 14: Breaker plus 14 days
WDR: WD40-repeat
ERF: Ethylene responsive factor
PAP1/PAP: Production of anthocyanin pigment1/production of anthocyanin pigment
TT2: Transparent testa2
AP2/ERF: Apetala2/ethylene response factor
PIF3: Phytochrome interacting factor 3
TT8: Transparent testa8
GL3: GLABRA3
EGL3: Enhancer of glabra3
Y2H: Yeast two-hybrid assays
PPFD: photosynthetic photon flux density

## References

[1] Zhu Z, Sun B, Cai W, Zhou X, Mao Y, Chen C, Wei J, Cao B, Chen C, Chen G, Lei J (2019). Natural variations in the MYB transcription factor *MYB31* determine the evolution of extremely pungent peppers. New Phytol.

[2] Sharma SK, Vij AS, Sharma M (2013). Mechanisms and clinical uses of capsaicin. Eur J Pharmacol.

[3] Spiller F, Alves MK, Vieira SM, Carvalho TA, Leite CE, Lunardelli A, Poloni JA, Cunha FQ, de Oliveira JR (2008). Anti-inflammatory effects of red pepper (*Capsicum baccatum*) on carrageenan- and antigen-induced inflammation. J Pharm Pharmacol.

[4] Melendez-Martinez AJ (2019). An overview of carotenoids, apocarotenoids, and vitamin A in agro-food, nutrition, health, and disease. Mol Nutr Food Res.

[5] Gomes LM, Petito N, Costa VG, Falcao DQ, de Lima AK (2014). Inclusion complexes of red bell pepper pigments with beta-cyclodextrin: preparation, characterisation and application as natural colorant in yogurt. Food Chem.

[6] Berry HM, Rickett DV, Baxter CJ, Enfissi E, Fraser PD (2019). Carotenoid biosynthesis and sequestration in red chilli pepper fruit and its impact on colour intensity traits. J Eep Bot.

[7] Guzman I, Hamby S, Romero J, Bosland PW, O’Connell MA (2010). Variability of carotenoid biosynthesis in orange colored *Capsicum* spp. Plant Sci.

[8] Fujiwake H, Suzuki T, Iwai K (2006). Intracellular localization of capsaicin and its analogues in *Capsicum* fruit II. The vacuole as the intracellular accumulation site of capsaicinoid in the protoplast of *Capsicum* fruit. Plant Cell Physiol.

[9] Reeves G, Bosland PW, Coon D (2012). ‘Trinidad Moruga Scorpion’ pepper is the world’s hottest measured chile pepper at more than two million scoville heat units. Horttechnology.

[10] Carle R, Schieber A, Schweiggert U (2006). Characterization of major and minor capsaicinoids and related compounds in chili pods (*Capsicum frutescens* L.) by high-performance liquid chromatography/atmospheric pressure chemical ionization mass spectrometry. Anal Chim Acta.

[11] Stewart CJ, Kang BC, Liu K, Mazourek M, Moore SL, Yoo EY, Kim BD, Paran I, Jahn MM (2005). The *Pun1* gene for pungency in pepper encodes a putative acyltransferase. Plant J.

[12] Sun B, Zhu Z, Chen C, Chen G, Cao B, Chen C, Lei J (2019). Jasmonate-inducible R2R3-MYB transcription factor regulates capsaicinoid biosynthesis and stamen development in *Capsicum*. J Agric Food Chem.

[13] Choi H, Yu Y, Jo YD, Ahn HK, Park B, Yeam I, Kim K, Park J, Choi YD, Park M (2014). Genome sequence of the hot pepper provides insights into the evolution of pungency in *Capsicum* species. Nat Genet.

[14] Ledent V, Vervoort M (2001). The basic helix-loop-helix protein family: comparative genomics and phylogenetic analysis. Genome Res.

[15] Toledo-Ortiz G, Huq E, Quail PH (2003). The *Arabidopsis* basic/helix-loop-helix transcription factor family. Plant Cell.

[16] Murre C, McCaw PS, Baltimore D (1989). A new DNA binding and dimerization motif in immunoglobulin enhancer binding, daughterless, MyoD, and myc proteins. Cell.

[17] Atchley WR, Terhalle W, Dress A (1999). Positional dependence, cliques, and predictive motifs in the bHLH protein domain. J Mol Evol.

[18] Massari ME, Murre C (2000). Helix-loop-helix proteins: regulators of transcription in eucaryotic organisms. Mol Cell Biol.

[19] Ferre-D'Amare AR, Pognonec P, Roeder RG, Burley SK (1994). Structure and function of the b/HLH/Z domain of USF. Embo J.

[20] Morgenstern B, Atchley WR (1999). Evolution of bHLH transcription factors: modular evolution by domain shuffling?. Mol Biol Evol.

[21] Heim MA, Jakoby M, Werber M, Martin C, Weisshaar B, Bailey PC (2003). The basic helix-loop-helix transcription factor family in plants: a genome-wide study of protein structure and functional diversity. Mol Biol Evol.

[22] Li X, Duan X, Jiang H, Sun Y, Tang Y, Yuan Z, Guo J, Liang W, Chen L, Yin J (2006). Genome-wide analysis of basic/helix-loop-helix transcription factor family in rice and *Arabidopsis*. Plant Physiol.

[23] Carretero-Paulet L, Galstyan A, Roig-Villanova I, Martinez-Garcia JF, Bilbao-Castro JR, Robertson DL (2010). Genome-wide classification and evolutionary analysis of the bHLH family of transcription factors in *Arabidopsis*, poplar, rice, moss, and algae. Plant Physiol.

[24] Wang R, Zhao P, Kong N, Lu R, Pei Y, Huang C, Ma H, Chen Q (2018). Genome-wide identification and characterization of the potato bHLH transcription factor family. Genes.

[25] Wang P, Su L, Gao H, Jiang X, Wu X, Li Y, Zhang Q, Wang Y, Ren F (2018). Genome-wide characterization of bHLH genes in grape and analysis of their potential relevance to abiotic stress tolerance and secondary metabolite biosynthesis. Front Plant Sci.

[26] Cui X, Wang YX, Liu ZW, Wang WL, Li H, Zhuang J (2018). Transcriptome-wide identification and expression profile analysis of the bHLH family genes in *Camellia sinensis*. Funct Integr Genomics.

[27] Song XM, Huang ZN, Duan WK, Ren J, Liu TK, Li Y, Hou XL (2014). Genome-wide analysis of the bHLH transcription factor family in Chinese cabbage (*Brassica rapa* ssp*. pekinensis*). Mol Genet Genomics.

[28] Zhang T, Lv W, Zhang H, Ma L, Li P, Ge L, Li G (2018). Genome-wide analysis of the basic Helix-Loop-Helix (bHLH) transcription factor family in maize. BMC Plant Biol.

[29] Wang J, Hu Z, Zhao T, Yang Y, Chen T, Yang M, Yu W, Zhang B (2015). Genome-wide analysis of bHLH transcription factor and involvement in the infection by yellow leaf curl virus in tomato (*Solanum lycopersicum*). BMC Genomics.

[30] Sun H, Fan HJ, Ling HQ (2015). Genome-wide identification and characterization of the bHLH gene family in tomato. BMC Genomics.

[31] Diao W, Snyder JC, Wang S, Liu J, Pan B, Guo G, Ge W, Dawood M (2018). Genome-wide analyses of the NAC transcription factor gene family in pepper (*Capsicum annuum* L*.*): chromosome location, phylogeny, structure, expression profiles, cis-elements in the promoter, and interaction network. Int J Mol Sci.

[32] Jin JH, Wang M, Zhang HX, Khan A, Wei AM, Luo DX, Gong ZH (2018). Genome-wide identification of the AP2/ERF transcription factor family in pepper (*Capsicum annuum* L*.*). Genome.

[33] Cheng Y, Yao ZP, Ruan MY, Ye QJ, Wang RQ, Zhou GZ, Luo J. In silico identification and characterization of the WRKY gene superfamily in pepper (*Capsicum annuum* L*.*). Genet Mol Res. 2016;15(3):1–12. 10.4238/gmr.15038675 27706772

[34] Song J, Chen C, Zhang S, Wang J, Huang Z, Chen M, Cao B, Zhu Z, Lei J (2020). Systematic analysis of the *Capsicum* ERF transcription factor family: identification of regulatory factors involved in the regulation of species-specific metabolites. BMC Genomics.

[35] Shin J, Park E, Choi G (2007). PIF3 regulates anthocyanin biosynthesis in an HY5-dependent manner with both factors directly binding anthocyanin biosynthetic gene promoters in *Arabidopsis*. Plant J.

[36] Park J, Nou I, Kim H, Afrin KS, Kang S, Rahim MA, Jung H (2018). Expression of anthocyanin biosynthesis-related genes reflects the peel color in purple tomato. Hortic Environ Biote.

[37] Zhu Z, Chen G, Guo X, Yin W, Yu X, Hu J, Hu Z (2017). Overexpression of *SlPRE2*, an atypical bHLH transcription factor, affects plant morphology and fruit pigment accumulation in tomato. Sci Rep.

[38] Llorente B, D'Andrea L, Ruiz-Sola MA, Botterweg E, Pulido P, Andilla J, Loza-Alvarez P, Rodriguez-Concepcion M (2016). Tomato fruit carotenoid biosynthesis is adjusted to actual ripening progression by a light-dependent mechanism. Plant J.

[39] Shen X, Zhao K, Liu L, Zhang K, Yuan H, Liao X, Wang Q, Guo X, Li F, Li T (2014). A role for PacMYBA in ABA-regulated anthocyanin biosynthesis in red-colored sweet cherry cv. Hong Deng (*Prunus avium* L*.*). Plant Cell Physiol.

[40] Lu B, Cheng G, Zhang Z, Sun J, Ali M, Jia Q, Luo D, Gong Z, Li D (2019). CaMYC, a novel transcription factor, regulates anthocyanin biosynthesis in color-leaved pepper (*Capsicum annuum* L*.*). J Plant Growth Regul.

[41] Cardenas PD, Sonawane PD, Pollier J, Vanden BR, Dewangan V, Weithorn E, Tal L, Meir S, Rogachev I, Malitsky S (2016). GAME9 regulates the biosynthesis of steroidal alkaloids and upstream isoprenoids in the plant mevalonate pathway. Nat Commun.

[42] Zhang H, Hedhili S, Montiel G, Zhang Y, Chatel G, Pre M, Gantet P, Memelink J (2011). The basic helix-loop-helix transcription factor CrMYC2 controls the jasmonate-responsive expression of the *ORCA* genes that regulate alkaloid biosynthesis in *Catharanthus roseus*. Plant J.

[43] De Boer K, Tilleman S, Pauwels L, Vanden BR, De Sutter V, Vanderhaeghen R, Hilson P, Hamill JD, Goossens A (2011). APETALA2/ETHYLENE RESPONSE FACTOR and basic helix-loop-helix tobacco transcription factors cooperatively mediate jasmonate-elicited nicotine biosynthesis. Plant J.

[44] Baudry A, Heim MA, Dubreucq B, Caboche M, Weisshaar B, Lepiniec L (2004). TT2, TT8, and TTG1 synergistically specify the expression of *BANYULS* and proanthocyanidin biosynthesis in *Arabidopsis thaliana*. Plant J.

[45] Zimmermann IM, Heim MA, Weisshaar B, Uhrig JF (2004). Comprehensive identification of *Arabidopsis thaliana* MYB transcription factors interacting with R/B-like BHLH proteins. Plant J.

[46] Butelli E, Titta L, Giorgio M, Mock HP, Matros A, Peterek S, Schijlen EG, Hall RD, Bovy AG, Luo J (2008). Enrichment of tomato fruit with health-promoting anthocyanins by expression of select transcription factors. Nat Biotechnol.

[47] Grotewold E, Sainz MB, Tagliani L, Hernandez JM, Bowen B, Chandler VL (2000). Identification of the residues in the Myb domain of maize C1 that specify the interaction with the bHLH cofactor R. Proc Natl Acad Sci U S A.

[48] Feller A, Machemer K, Braun EL, Grotewold E (2011). Evolutionary and comparative analysis of MYB and bHLH plant transcription factors. Plant J.

[49] Goff SA, Cone KC, Chandler VL (1992). Functional analysis of the transcriptional activator encoded by the maize B gene: evidence for a direct functional interaction between two classes of regulatory proteins. Genes Dev.

[50] Arce-Rodriguez ML, Ochoa-Alejo N (2017). An R2R3-MYB transcription factor regulates capsaicinoid biosynthesis. Plant Physiol.

[51] Sun B, Zhou X, Chen C, Chen C, Chen K, Chen M, Liu S, Chen G, Cao B, Cao F, Lei J. Coexpression network analysis reveals an MYB transcriptional activator involved in capsaicinoid biosynthesis in hot peppers. Hortic Res-England. 2020;7(162):1–14. 10.1038/s41438-020-00381-2 PMC7527512 33082969

[52] Kim S, Park M, Yeom SI, Kim YM, Lee JM, Lee HA, Seo E, Choi J, Cheong K, Kim KT, Jung K, Lee GW, Oh SK, Bae C, Kim SB, Lee HY, Kim SY, Kim MS, Kang BC, Jo YD, Yang HB, Jeong HJ, Kang WH, Kwon JK, Shin C, Lim JY, Park JH, Huh JH, Kim JS, Kim BD, Cohen O, Paran I, Suh MC, Lee SB, Kim YK, Shin Y, Noh SJ, Park J, Seo YS, Kwon SY, Kim HA, Park JM, Kim HJ, Choi SB, Bosland PW, Reeves G, Jo SH, Lee BW, Cho HT, Choi HS, Lee MS, Yu Y, Do Choi Y, Park BS, van Deynze A, Ashrafi H, Hill T, Kim WT, Pai HS, Ahn HK, Yeam I, Giovannoni JJ, Rose JK, Sørensen I, Lee SJ, Kim RW, Choi IY, Choi BS, Lim JS, Lee YH, Choi D (2014). Genome sequence of the hot pepper provides insights into the evolution of pungency in *Capsicum* species. Nat Genet.

[53] Naves ER, de Avila SL, Sulpice R, Araujo WL, Nunes-Nesi A, Peres L, Zsogon A (2019). Capsaicinoids: pungency beyond *Capsicum*. Trends Plant Sci.

[54] Dong X, Yan Y, Jiang B, Shi Y, Jia Y, Cheng J, Shi Y, Kang J, Li H, Zhang D, Qi L, Han R, Zhang S, Zhou Y, Wang X, Terzaghi W, Gu H, Kang D, Yang S, Li J (2020). The cold response regulator CBF1 promotes *Arabidopsis* hypocotyl growth at ambient temperatures. EMBO J.

[55] Lescot M, Dehais P, Thijs G, Marchal K, Moreau Y, Van de Peer Y, Rouze P, Rombauts S (2002). PlantCARE, a database of plant cis-acting regulatory elements and a portal to tools for in silico analysis of promoter sequences. Nucleic Acids Res.

[56] Hudson KA, Hudson ME (2015). A classification of basic helix-loop-helix transcription factors of soybean. Int J Genomics..

[57] Mao K, Dong Q, Li C, Liu C, Ma F (2017). Genome wide identification and characterization of apple bHLH transcription factors and expression analysis in response to drought and salt stress. Front Plant Sci.

[58] Atchley WR, Fitch WM (1997). A natural classification of the basic helix-loop-helix class of transcription factors. Proc Natl Acad Sci U S A.

[59] Shimizu T, Toumoto A, Ihara K, Shimizu M, Kyogoku Y, Ogawa N, Oshima Y, Hakoshima T (1997). Crystal structure of PHO4 bHLH domain-DNA complex: flanking base recognition. EMBO J.

[60] Yue H, Wang M, Liu S, Du X, Song W, Nie X (2016). Transcriptome-wide identification and expression profiles of the WRKY transcription factor family in Broomcorn millet (*Panicum miliaceum L.*). BMC Genomics.

[61] Frerigmann H, Berger B, Gigolashvili T (2014). bHLH05 is an interaction partner of MYB51 and a novel regulator of glucosinolate biosynthesis in *Arabidopsis*. Plant Physiol.

[62] Abe H, Urao T, Ito T, Seki M, Shinozaki K, Yamaguchi-Shinozaki K (2003). *Arabidopsis* AtMYC2 (bHLH) and AtMYB2 (MYB) function as transcriptional activators in abscisic acid signaling. Plant Cell.

[63] Lorenzo O, Chico JM, Sanchez-Serrano JJ, Solano R (2004). JASMONATE-INSENSITIVE1 encodes a MYC transcription factor essential to discriminate between different jasmonate-regulated defense responses in *Arabidopsis*. Plant Cell.

[64] Yadav V, Mallappa C, Gangappa SN, Bhatia S, Chattopadhyay S (2005). A basic helix-loop-helix transcription factor in *Arabidopsis*, MYC2, acts as a repressor of blue light-mediated photomorphogenic growth. Plant Cell.

[65] Nesi N, Debeaujon I, Jond C, Pelletier G, Caboche M, Lepiniec L (2000). The *TT8* gene encodes a basic helix-loop-helix domain protein required for expression of *DFR* and *BAN* genes in *Arabidopsis* siliques. Plant Cell.

[66] Payne CT, Zhang F, Lloyd AM (2000). *GL3* encodes a bHLH protein that regulates trichome development in *Arabidopsis* through interaction with GL1 and TTG1. Genetics.

[67] Bernhardt C, Lee MM, Gonzalez A, Zhang F, Lloyd A, Schiefelbein J (2003). The bHLH genes *GLABRA3 (GL3)* and *ENHANCER OF GLABRA3 (EGL3)* specify epidermal cell fate in the *Arabidopsis* root. Development.

[68] Xu J, van Herwijnen ZO, Drager DB, Sui C, Haring MA, Schuurink RC (2018). SlMYC1 regulates type VI glandular trichome formation and terpene biosynthesis in tomato glandular cells. Plant Cell.

[69] Feng HL, Ma NN, Meng X, Zhang S, Wang JR, Chai S, Meng QW (2013). A novel tomato MYC-type ICE1-like transcription factor, SlICE1a, confers cold, osmotic and salt tolerance in transgenic tobacco. Plant Physiol Biochem.

[70] Leivar P, Quail PH (2011). PIFs: pivotal components in a cellular signaling hub. Trends Plant Sci.

[71] Keyhaninejad N, Curry J, Romero J, O'Connell MA (2014). Fruit specific variability in capsaicinoid accumulation and transcription of structural and regulatory genes in *Capsicum* fruit. Plant Sci.

[72] Koini MA, Alvey L, Allen T, Tilley CA, Harberd NP, Whitelam GC, Franklin KA (2009). High temperature-mediated adaptations in plant architecture require the bHLH transcription factor PIF4. Curr Biol.

[73] Qin C, Yu C, Shen Y, Fang X, Chen L, Min J, Cheng J, Zhao S, Xu M, Luo Y, Yang Y, Wu Z, Mao L, Wu H, Ling-Hu C, Zhou H, Lin H, González-Morales S, Trejo-Saavedra DL, Tian H, Tang X, Zhao M, Huang Z, Zhou A, Yao X, Cui J, Li W, Chen Z, Feng Y, Niu Y, Bi S, Yang X, Li W, Cai H, Luo X, Montes-Hernández S, Leyva-González MA, Xiong Z, He X, Bai L, Tan S, Tang X, Liu D, Liu J, Zhang S, Chen M, Zhang L, Zhang L, Zhang Y, Liao W, Zhang Y, Wang M, Lv X, Wen B, Liu H, Luan H, Zhang Y, Yang S, Wang X, Xu J, Li X, Li S, Wang J, Palloix A, Bosland PW, Li Y, Krogh A, Rivera-Bustamante RF, Herrera-Estrella L, Yin Y, Yu J, Hu K, Zhang Z (2014). Whole-genome sequencing of cultivated and wild peppers provides insights into *Capsicum* domestication and specialization. Proc Natl Acad Sci U S A.

[74] El-Gebali S, Mistry J, Bateman A, Eddy SR, Luciani A, Potter SC, Qureshi M, Richardson LJ, Salazar GA, Smart A, Sonnhammer E, Hirsh L, Paladin L, Piovesan D, Tosatto S, Finn R (2019). The Pfam protein families database in 2019. Nucleic Acids Res.

[75] Potter SC, Luciani A, Eddy SR, Park Y, Lopez R, Finn RD (2018). HMMER web server: 2018 update. Nucleic Acids Res.

[76] Letunic I, Bork P (2018). 20 years of the SMART protein domain annotation resource. Nucleic Acids Res.

[77] Lu S, Wang J, Chitsaz F, Derbyshire MK, Geer RC, Gonzales NR, Gwadz M, Hurwitz DI, Marchler GH, Song JS, Thanki N, Yamashita RA, Yang MZ, Zhang DC, Zheng CJ, Lanczycki CJ, Marchler-Bauer A (2020). CDD/SPARCLE: the conserved domain database in 2020. Nucleic Acids Res.

[78] Gasteiger E, Gattiker A, Hoogland C, Ivanyi I, Appel RD, Bairoch A (2003). ExPASy: The proteomics server for in-depth protein knowledge and analysis. Nucleic Acids Res.

[79] Lamesch P, Berardini TZ, Li D, Swarbreck D, Wilks C, Sasidharan R, Muller R, Dreher K, Alexander DL, Garcia-Hernandez M, Karthikeyan AS, Lee CH, Nelson WD, Ploetz L, Singh S, Wensel A, Hula E (2012). The Arabidopsis Information Resource (TAIR): improved gene annotation and new tools. Nucleic Acids Res.

[80] Tian F, Yang DC, Meng YQ, Jin J, Gao G (2020). PlantRegMap: charting functional regulatory maps in plants. Nucleic Acids Res.

[81] Kumar S, Stecher G, Li M, Knyaz C, Tamura K (2018). MEGA X: molecular evolutionary genetics analysis across computing platforms. Mol Biol Evol.

[82] Subramanian B, Gao S, Lercher MJ, Hu S, Chen WH (2019). Evolview v3: a webserver for visualization, annotation, and management of phylogenetic trees. Nucleic Acids Res.

[83] Bailey TL, Boden M, Buske FA, Frith M, Grant CE, Clementi L, Ren J, Li WW, Noble WS (2009). MEME SUITE: tools for motif discovery and searching. Nucleic Acids Res.

[84] Trapnell C, Williams BA, Pertea G, Mortazavi A, Kwan G, van Baren MJ, Salzberg SL, Wold BJ, Pachter L (2010). Transcript assembly and quantification by RNA-Seq reveals unannotated transcripts and isoform switching during cell differentiation. Nat Biotechnol.

[85] Trapnell C, Pachter L, Salzberg SL (2009). TopHat: discovering splice junctions with RNA-Seq. Bioinformatics.

[86] Wang Y, Chen G, Lei J, Cao B, Chen C (2020). Identification and characterization of a LEA-like gene, *CaMF5*, specifically expressed in the anthers of male-fertile *Capsicum annuum*. Horticultural Plant J.

[87] Livak KJ, Schmittgen TD (2001). Analysis of relative gene expression data using real-time quantitative PCR and the 2(-Delta Delta C(T)) method. Methods.

