# Supplementary material for: Genome-wide identification of the Capsicum bHLH transcription factor family: discovery of a candidate regulator involved in the regulation of species-specific bioactive metabolites
Source: BMC Plant Biol. 2021 Jun 7;21:262. doi: 10.1186/s12870-021-03004-7 (PMC8183072; doi:10.1186/s12870-021-03004-7)
Supplement: Supplementary file 1 — Additional file 1: Figure S1. Carotenoid (A) and capsaicinoid (B) biosynthetic pathways. Figure S2. Chromosomal localizations of CabHLHs. Figure S3. Multiple alignment analysis of the bHLH domain in pepper bHLH proteins. Figure S4. Phylogenetic tree of the bHLH family in Capsicum annuum, Arabidopsis thaliana, tomato and rice. Figure S5. The expression profiles of CabHLH genes in different tissues. Figure S6. Diagram of yeast two-hybrid assays experiments [file 12870_2021_3004_MOESM1_ESM.pdf]

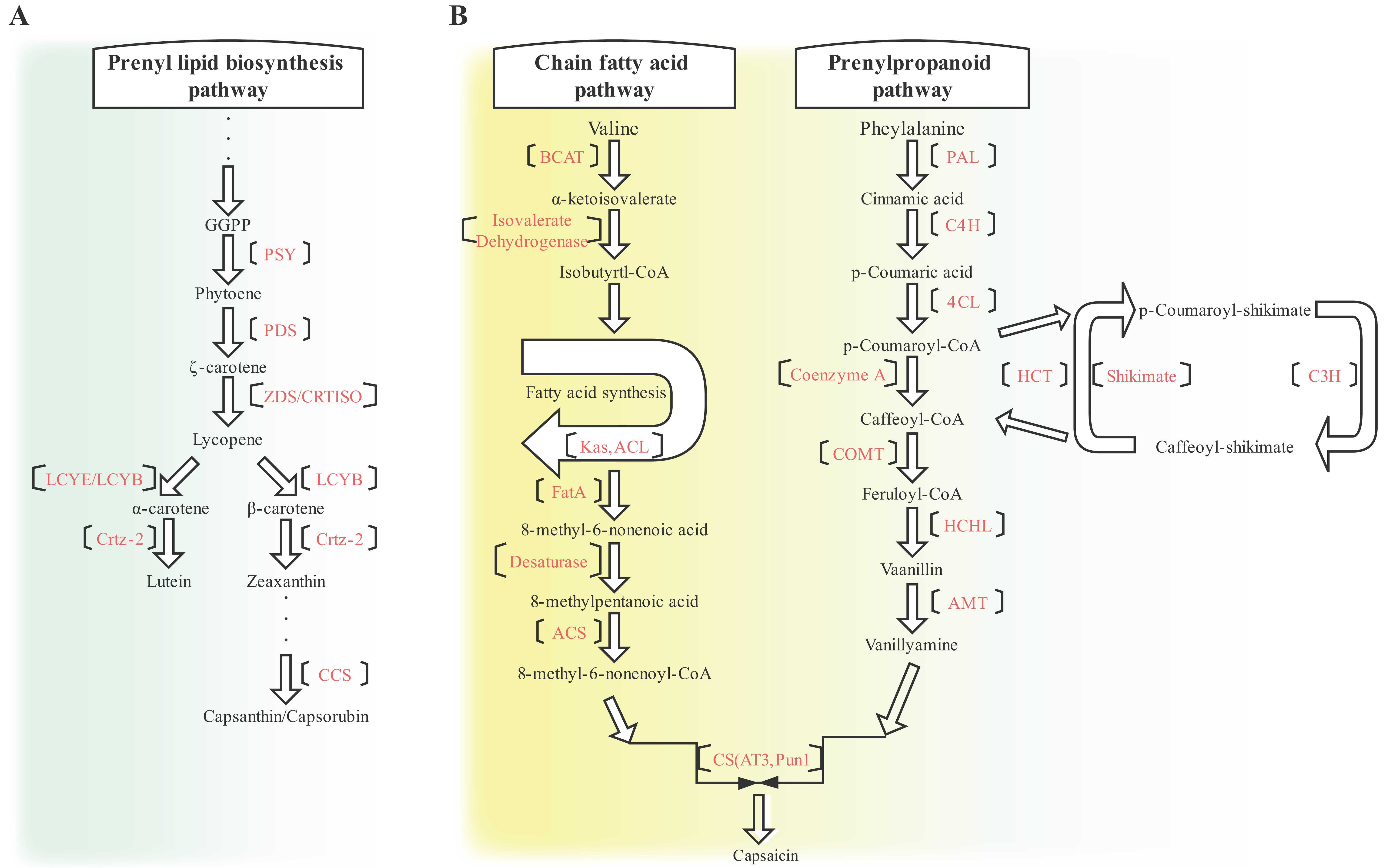

Figure S1. Carotenoid (A) and capsaicinoid (B) biosynthetic pathways. A. In the first step, Geranylgeranyl pyrophosphate (GGPP) is catalyzed by a series of synthetases, and transformed to lycopene. Then, there are two different branches: α-carotene finally is developed into lutein, and β-carotene is catalyzed to transform into capsorubin or capsanthin by capsanthin/capsorubin synthase (CCS). The ellipsis indicates intermediate steps. B. Phenylalanine and chain fatty acid biosynthesis pathways which are adjusted by a series of genes encoding enzymes synthesize capsaicin together.

ZDS, ζ-carotene desaturase; CRTISO, carotenoids isomerase; LCYE, lycopene ε-cyclase; CrtZ-2, β-carotene hydroxylase-2; PAL, phenylalanine ammonia-lyase; C4H, cinnamate 4-hydroxylase; 4CL, 4-coumaroyl-CoA ligase; HCT, hydroxycinnamoyl transferase; C3H, p-coumaroyl shikimate/quinate 3-hydroxylase; COMT, caffeoyl-CoA 3-O-methyltransferase; HCHL, hydroxycinnamoyl-CoA hydratase lyase; AMT, aminotransferase;
BCAT, branched-chain amino acid aminotransferase; Kas, ketoacyl-ACP synthase; ACL, acyl carrier protein; and FatA, acyl-ACP thioesterase.

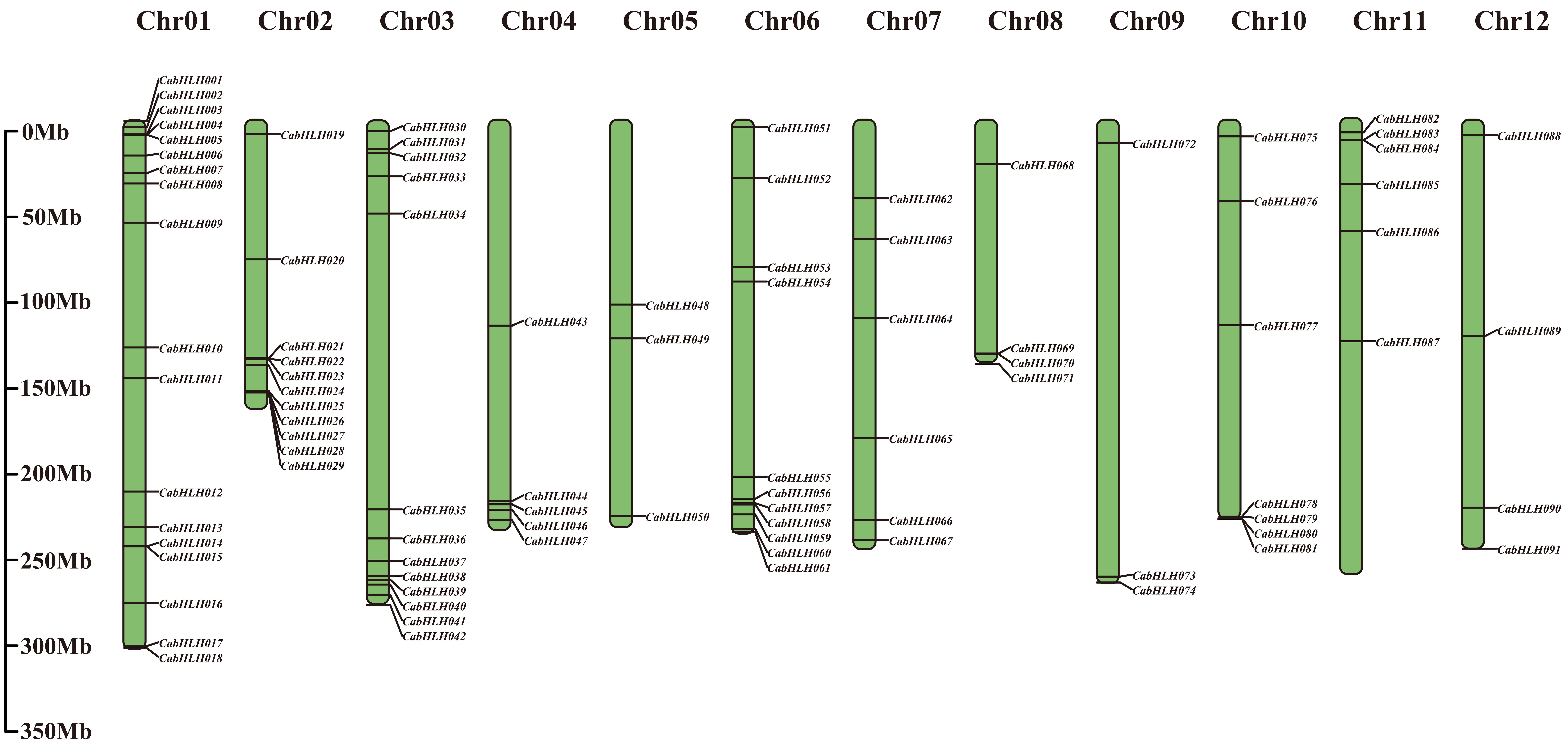

Figure S2. Chromosomal localizations of *CabHLH*s.The chromosome distribution of each *CabHLH* was obtained from the 2.0 version *Capsicum annuum* genome annotation information, and TBtools (1.068 version) were used to make the linkage visualization.

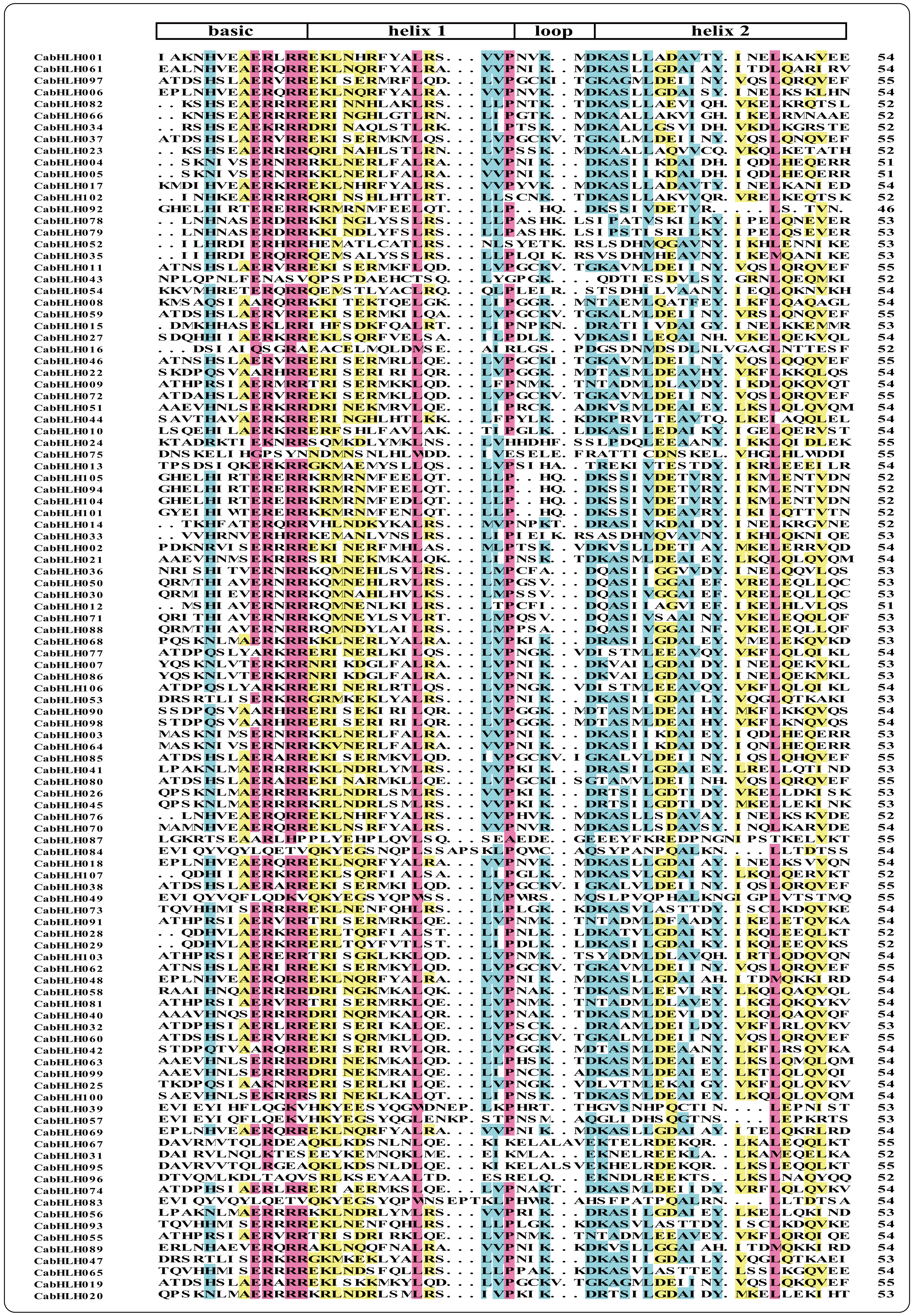

Figure S3. Multiple alignment analysis of the bHLH domain in pepper bHLH proteins. Multiple alignments were performed by Clustal X 2.1 with the default parameters. Amino acids with greater than 75% are shown in pink, those with 50 to 75% identity are shown in cyan, and those with 33 to 50% identity are shown in yellow. Dotted lines indicate gaps.

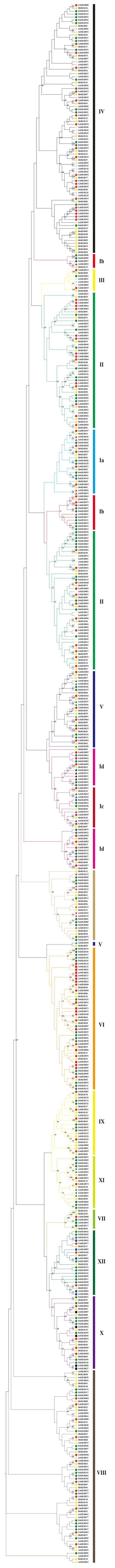

Figure S4. Phylogenetic tree of the bHLH family in *Capsicum annuum*, *Arabidopsis thaliana*, tomato, and rice. The different-coloured branches indicated different groups of bHLH proteins. Red, yellow and green circles represented bHLH proteins in *Capsicum annuum*, tomato and rice, respectively. Other different colours and shapes indicated different groups in phylogeny of *Arabidopsis thaliana*.

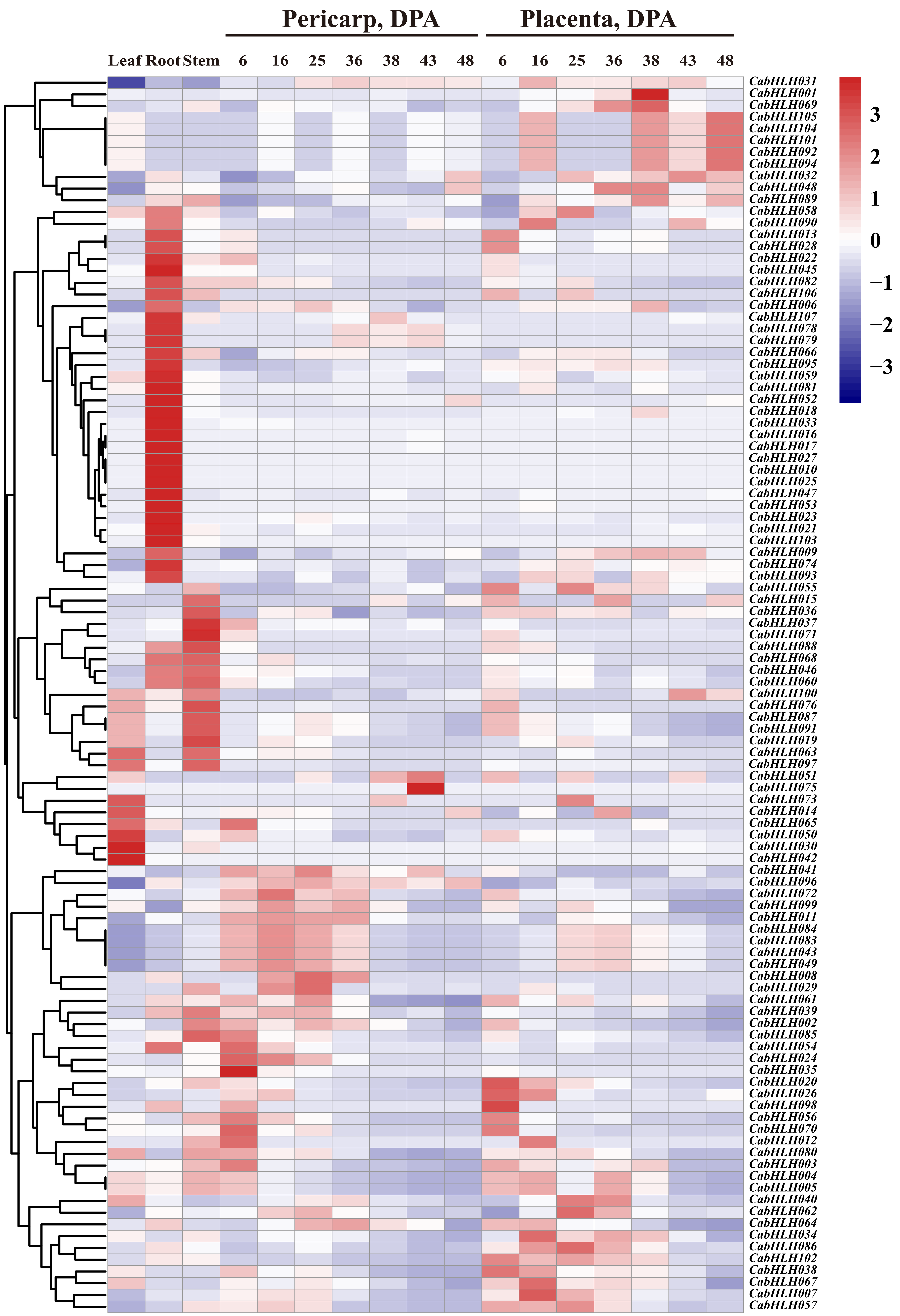

Figure S5. The expression patterns of *CabHLH* genes in different tissues. The RPKM values which was mapped to the *Capsicum annuum* genome of 1.5 version were performed by Kim et al (2014) and used to construct the heat map.

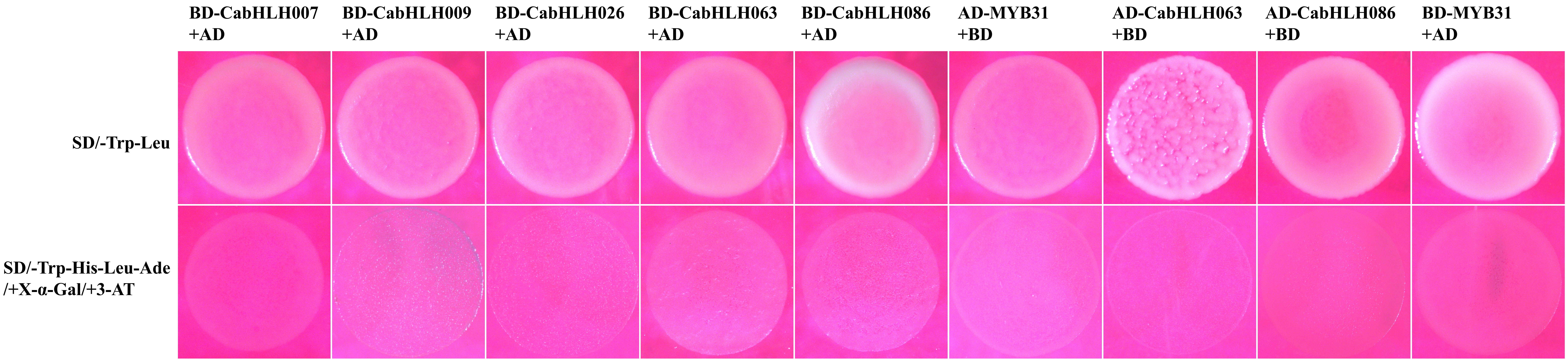

Figure S6. Diagram of yeast two-hybrid assays experiment. AD and BD represent empty pGADT7 and pGBKT7 vectors, separately. Among them, the self-activation function of CabHLH009 and CabHLH026 were inhibited under 15mMol and 30mMol 3-AT, separately. The rest lacked the self-activation function.
