# Supplementary material for: Genome-wide identification of the Capsicum bHLH transcription factor family: discovery of a candidate regulator involved in the regulation of species-specific bioactive metabolites
Source: BMC Plant Biol. 2021 Jun 7;21:262. doi: 10.1186/s12870-021-03004-7 (PMC8183072; doi:10.1186/s12870-021-03004-7)
Supplement: Supplementary file 2 — Additional file 2: Table S1. The corresponding information of 107 CabHLHs. Table S2. Functionally characterized partial bHLH proteins from tomato and Arabidopsis thaliana. Table S3. Features of the CabHLH proteins motifs. Table S4. List of the bHLH genes in rice, tomato and Arabidopsis thaliana. Table S5. List of the primer for real-time quantitative PCR. Table S6. List of the primer for yeast two-hybrid assays. Table S7. Putative cis-elements of capsorubin and capsaicin biosynthetic genes promoters. [file 12870_2021_3004_MOESM2_ESM.doc]

**Table S1. The corresponding information of 107 *CabHLH*s**

|  | **Chr** | **Position** | **Length** | **MW** | **pI** | **Instability** | **Corresponding gene ID** |
| --- | --- | --- | --- | --- | --- | --- | --- |
|  |  |  | **(aa)** | **(kDa)** |  | **index (II)** | **in CM334** |
| CabHLH001 | Chr01 | 448960-450264 | 434 | 49.07 | 6.68 | 45.50 | CA.PGAv.1.6.scaffold567.91 |
| CabHLH002 | Chr01 | 3999289-4002858 | 612 | 68.77 | 5.86 | 52.21 | CA.PGAv.1.6.scaffold774.51 |
| CabHLH003 | Chr01 | 8068888-8070165 | 256 | 29.43 | 5.59 | 71.81 | CA.PGAv.1.6.scaffold630.34 |
| CabHLH004 | Chr01 | 8405925-8409372 | 190 | 21.76 | 4.78 | 99.37 | CA.PGAv.1.6.scaffold630.47 |
| CabHLH005 | Chr01 | 8427511-8431651 | 247 | 28.00 | 5.15 | 82.57 | CA.PGAv.1.6.scaffold630.49 |
| CabHLH006 | Chr01 | 20556324-20558396 | 690 | 75.44 | 5.54 | 48.67 | CA.PGAv.1.6.scaffold298.8 |
| CabHLH007 | Chr01 | 30841525-30844271 | 480 | 53.59 | 5.59 | 38.19 | CA.PGAv.1.6.scaffold1731.6 |
| CabHLH008 | Chr01 | 36854403-36855872 | 348 | 39.73 | 4.88 | 56.95 | CA.PGAv.1.6.scaffold22.31 |
| CabHLH009 | Chr01 | 59674270-59676435 | 394 | 43.33 | 5.64 | 52.14 | CA.PGAv.1.6.scaffold170.131 |
| CabHLH010 | Chr01 | 132461364-132464145 | 270 | 30.11 | 6.22 | 52.14 | CA.PGAv.1.6.scaffold14.58 |
| CabHLH011 | Chr01 | 150346085-150348543 | 542 | 60.28 | 7.09 | 54.00 | CA.PGAv.1.6.scaffold366.7 |
| CabHLH012 | Chr01 | 216454825-216456972 | 193 | 21.61 | 9.02 | 63.23 | CA.PGAv.1.6.scaffold183.18 |
| CabHLH013 | Chr01 | 237201263-237202988 | 223 | 25.05 | 7.64 | 67.90 | CA.PGAv.1.6.scaffold556.6 |
| CabHLH014 | Chr01 | 248435644-248446770 | 940 | 106.70 | 5.88 | 48.01 | CA.PGAv.1.6.scaffold700.27 |
| CabHLH015 | Chr01 | 248451138-248456337 | 510 | 58.52 | 5.79 | 49.21 | CA.PGAv.1.6.scaffold700.26 |
| CabHLH016 | Chr01 | 281412337-281417061 | 325 | 36.18 | 4.65 | 62.68 | CA.PGAv.1.6.scaffold346.13 |
| CabHLH017 | Chr01 | 306522034-306524208 | 426 | 48.88 | 5.48 | 48.82 | CA.PGAv.1.6.scaffold792.24 |
| CabHLH018 | Chr01 | 307837320-307839164 | 614 | 66.89 | 5.28 | 50.52 | CA.PGAv.1.6.scaffold1216.16 |
| CabHLH019 | Chr02 | 8341910-8343354 | 348 | 39.15 | 7.12 | 52.04 | CA.PGAv.1.6.scaffold53.18 |
| CabHLH020 | Chr02 | 81468229-81471032 | 330 | 37.06 | 5.21 | 51.21 | CA.PGAv.1.6.scaffold215.5 |
| CabHLH021 | Chr02 | 139204458-139207884 | 395 | 43.30 | 5.12 | 59.72 | CA.PGAv.1.6.scaffold370.46 |
| CabHLH022 | Chr02 | 139352427-139353089 | 220 | 24.52 | 8.93 | 77.40 | CA.PGAv.1.6.scaffold370.57 |
| CabHLH023 | Chr02 | 139635673-139638067 | 246 | 27.16 | 8.42 | 47.75 | CA.PGAv.1.6.scaffold370.66 |
| CabHLH024 | Chr02 | 143112973-143115115 | 205 | 23.08 | 8.74 | 53.77 | CA.PGAv.1.6.scaffold1164.47 |
| CabHLH025 | Chr02 | 158411902-158420678 | 616 | 69.77 | 6.11 | 50.45 | CA.PGAv.1.6.scaffold1061.47 |
| CabHLH026 | Chr02 | 158799822-158801583 | 342 | 38.73 | 5.17 | 58.09 | CA.PGAv.1.6.scaffold1061.22 |
| CabHLH027 | Chr02 | 158982197-158984049 | 335 | 38.08 | 7.07 | 67.90 | CA.PGAv.1.6.scaffold1061.12 |
| CabHLH028 | Chr02 | 159005098-159006826 | 299 | 33.36 | 7.68 | 48.77 | CA.PGAv.1.6.scaffold1061.11 |
| CabHLH029 | Chr02 | 159052339-159060395 | 779 | 85.98 | 5.51 | 52.10 | CA.PGAv.1.6.scaffold1061.10 |
| CabHLH030 | Chr03 | 6324725-6327101 | 295 | 34.06 | 5.71 | 60.42 | CA.PGAv.1.6.scaffold500.9 |
| CabHLH031 | Chr03 | 16731443-16735885 | 225 | 25.93 | 5.23 | 60.19 | CA.PGAv.1.6.scaffold793.34 |
| CabHLH032 | Chr03 | 19108152-19113380 | 345 | 37.49 | 5.73 | 63.14 | CA.PGAv.1.6.scaffold626.5 |
| CabHLH033 | Chr03 | 32656400-32658115 | 295 | 33.96 | 8.70 | 63.09 | CA.PGAv.1.6.scaffold339.46 |
| CabHLH034 | Chr03 | 54314575-54315592 | 245 | 27.59 | 8.64 | 52.40 | CA.PGAv.1.6.scaffold156.9 |
| CabHLH035 | Chr03 | 226801517-226802803 | 248 | 28.35 | 9.20 | 56.58 | CA.PGAv.1.6.scaffold1283.1 |
| CabHLH036 | Chr03 | 243716055-243719056 | 367 | 39.78 | 5.73 | 62.55 | CA.PGAv.1.6.scaffold108.27 |
| CabHLH037 | Chr03 | 256703403-256705516 | 340 | 37.84 | 6.10 | 49.13 | CA.PGAv.1.6.scaffold637.53 |
| CabHLH038 | Chr03 | 265578719-265582272 | 270 | 29.66 | 8.63 | 61.52 | CA.PGAv.1.6.scaffold999.12 |
| CabHLH039 | Chr03 | 267835873-267839504 | 541 | 58.91 | 7.73 | 48.64 | CA.PGAv.1.6.scaffold982.35 |
| CabHLH040 | Chr03 | 270605319-270607270 | 368 | 41.04 | 8.07 | 70.67 | CA.PGAv.1.6.scaffold479.35 |
| CabHLH041 | Chr03 | 276695385-276698275 | 531 | 57.47 | 5.31 | 48.71 | CA.PGAv.1.6.scaffold843.20 |
| CabHLH042 | Chr03 | 282650067-282651287 | 406 | 45.04 | 5.95 | 50.01 | CA.PGAv.1.6.scaffold1143.34 |
| CabHLH043 | Chr04 | 120052087-120067140 | 355 | 40.23 | 5.98 | 54.78 | CA.PGAv.1.6.scaffold563.5 |
| CabHLH044 | Chr04 | 222432649-222434507 | 209 | 23.10 | 9.91 | 36.44 | CA.PGAv.1.6.scaffold475.37 |
| CabHLH045 | Chr04 | 224288762-224290401 | 351 | 39.24 | 4.60 | 57.03 | CA.PGAv.1.6.scaffold723.26 |
| CabHLH046 | Chr04 | 227423868-227426843 | 381 | 41.70 | 6.38 | 52.49 | CA.PGAv.1.6.scaffold416.1 |
| CabHLH047 | Chr04 | 233407010-233410376 | 324 | 36.68 | 5.26 | 44.45 | CA.PGAv.1.6.scaffold2082.3 |
| CabHLH048 | Chr05 | 107782681-107784459 | 592 | 65.62 | 7.59 | 37.41 | CA.PGAv.1.6.scaffold611.5 |
| CabHLH049 | Chr05 | 127524567-127546973 | 328 | 36.63 | 5.95 | 50.26 | CA.PGAv.1.6.scaffold73.10 |
| CabHLH050 | Chr05 | 230974336-230976917 | 407 | 45.93 | 5.09 | 60.08 | CA.PGAv.1.6.scaffold765.19 |
| CabHLH051 | Chr06 | 4358496-4367809 | 370 | 40.37 | 5.74 | 64.31 | CA.PGAv.1.6.scaffold941.44 |
| CabHLH052 | Chr06 | 33944148-33945620 | 223 | 25.98 | 8.66 | 52.68 | CA.PGAv.1.6.scaffold1101.2 |
| CabHLH053 | Chr06 | 85871970-85873694 | 324 | 36.59 | 5.10 | 47.87 | CA.PGAv.1.6.scaffold246.1 |
| CabHLH054 | Chr06 | 94366890-94368413 | 268 | 30.88 | 6.76 | 64.77 | CA.PGAv.1.6.scaffold28.4 |
| CabHLH055 | Chr06 | 208122902-208128769 | 257 | 28.11 | 7.02 | 54.27 | CA.PGAv.1.6.scaffold308.41 |
| CabHLH056 | Chr06 | 221007535-221010989 | 515 | 56.36 | 5.30 | 47.58 | CA.PGAv.1.6.scaffold423.44 |
| CabHLH057 | Chr06 | 223604809-223608541 | 587 | 64.72 | 9.07 | 57.67 | CA.PGAv.1.6.scaffold587.63 |
| CabHLH058 | Chr06 | 224381051-224383074 | 406 | 45.91 | 7.77 | 61.12 | CA.PGAv.1.6.scaffold587.21 |
| CabHLH059 | Chr06 | 230154563-230156789 | 305 | 34.13 | 7.72 | 49.60 | CA.PGAv.1.6.scaffold65.108 |
| CabHLH060 | Chr06 | 238681925-238683836 | 413 | 45.73 | 8.06 | 41.69 | CA.PGAv.1.6.scaffold874.21 |
| CabHLH061 | Chr06 | 240600554-240601987 | 477 | 52.89 | 6.41 | 39.77 | CA.PGAv.1.6.scaffold771.20 |
| CabHLH062 | Chr07 | 45772673-45776528 | 506 | 55.38 | 5.10 | 55.21 | CA.PGAv.1.6.scaffold182.6 |
| CabHLH063 | Chr07 | 69613778-69616312 | 513 | 56.81 | 6.74 | 65.82 | CA.PGAv.1.6.scaffold819.2 |
| CabHLH064 | Chr07 | 115702972-115710345 | 209 | 23.73 | 6.54 | 64.28 | CA.PGAv.1.6.scaffold33.15 |
| CabHLH065 | Chr07 | 185553606-185555981 | 517 | 58.31 | 7.22 | 58.41 | CA.PGAv.1.6.scaffold52.7 |
| CabHLH066 | Chr07 | 233311619-233314165 | 249 | 27.46 | 7.71 | 55.04 | CA.PGAv.1.6.scaffold456.24 |
| CabHLH067 | Chr07 | 245082354-245087434 | 241 | 26.59 | 7.70 | 48.57 | CA.PGAv.1.6.scaffold337.154 |
| CabHLH068 | Chr08 | 26053029-26057580 | 595 | 68.15 | 5.83 | 51.23 | CA.PGAv.1.6.scaffold432.3 |
| CabHLH069 | Chr08 | 136330270-136332087 | 605 | 67.36 | 6.91 | 43.40 | CA.PGAv.1.6.scaffold1415.24 |
| CabHLH070 | Chr08 | 136763023-136764426 | 467 | 51.41 | 5.50 | 41.80 | CA.PGAv.1.6.scaffold1045.42 |
| CabHLH071 | Chr08 | 142288160-142289986 | 340 | 38.61 | 5.03 | 56.35 | CA.PGAv.1.6.scaffold1134.25 |
| CabHLH072 | Chr09 | 13584513-13588438 | 472 | 52.97 | 5.75 | 47.19 | CA.PGAv.1.6.scaffold1325.4 |
| CabHLH073 | Chr09 | 266386468-266400627 | 576 | 65.23 | 6.21 | 42.54 | CA.PGAv.1.6.scaffold1583.1 |
| CabHLH074 | Chr09 | 269832265-269840219 | 287 | 30.76 | 6.16 | 48.30 | CA.PGAv.1.6.scaffold551.34 |
| CabHLH075 | Chr10 | 9777007-9782993 | 497 | 56.42 | 5.20 | 41.83 | CA.PGAv.1.6.scaffold578.14 |
| CabHLH076 | Chr10 | 47417257-47418705 | 482 | 53.11 | 5.89 | 37.69 | CA.PGAv.1.6.scaffold271.3 |
| CabHLH077 | Chr10 | 119912156-119914045 | 327 | 36.43 | 5.15 | 56.40 | CA.PGAv.1.6.scaffold95.15 |
| CabHLH078 | Chr10 | 231434459-231435856 | 253 | 28.41 | 5.56 | 41.67 | CA.PGAv.1.6.scaffold641.59 |
| CabHLH079 | Chr10 | 231438800-231440710 | 253 | 29.00 | 5.85 | 49.84 | CA.PGAv.1.6.scaffold641.58 |
| CabHLH080 | Chr10 | 232592861-232597935 | 355 | 38.97 | 5.67 | 49.20 | CA.PGAv.1.6.scaffold1161.5 |
| CabHLH081 | Chr10 | 232633259-232636560 | 378 | 41.20 | 8.94 | 48.63 | CA.PGAv.1.6.scaffold1161.6 |
| CabHLH082 | Chr11 | 8503793-8508099 | 301 | 33.05 | 6.05 | 54.41 | CA.PGAv.1.6.scaffold184.87 |
| CabHLH083 | Chr11 | 12990500-12997542 | 328 | 36.17 | 5.59 | 47.14 | CA.PGAv.1.6.scaffold677.14 |
| CabHLH084 | Chr11 | 13008529-13013422 | 327 | 36.30 | 5.15 | 55.32 | CA.PGAv.1.6.scaffold677.12 |
| CabHLH085 | Chr11 | 38523089-38528292 | 264 | 29.05 | 6.67 | 58.76 | CA.PGAv.1.6.scaffold27.57 |
| CabHLH086 | Chr11 | 66086073-66088810 | 480 | 53.69 | 5.42 | 42.09 | CA.PGAv.1.6.scaffold682.3 |
| CabHLH087 | Chr11 | 130299682-130310636 | 199 | 22.98 | 5.43 | 54.28 | CA.PGAv.1.6.scaffold331.6 |
| CabHLH088 | Chr12 | 8956140-8958630 | 325 | 37.23 | 8.13 | 64.05 | CA.PGAv.1.6.scaffold905.4 |
| CabHLH089 | Chr12 | 126157037-126158667 | 519 | 58.14 | 8.90 | 47.07 | CA.PGAv.1.6.scaffold382.7 |
| CabHLH090 | Chr12 | 226184436-226185910 | 252 | 28.25 | 9.86 | 69.39 | CA.PGAv.1.6.scaffold1194.4 |
| CabHLH091 | Chr12 | 250093080-250095621 | 449 | 49.20 | 5.22 | 53.15 | CA.PGAv.1.6.scaffold575.53 |
| CabHLH092 | Chr00 | 1481-4698 | 296 | 32.60 | 5.81 | 32.83 | CA.PGAv.1.6.scaffold1838.1 |
| CabHLH093 | Chr00 | 2087-5217 | 321 | 36.48 | 8.92 | 59.87 | CA.PGAv.1.6.scaffold3656.1 |
| CabHLH094 | Chr00 | 72161-76710 | 414 | 45.97 | 7.11 | 35.00 | CA.PGAv.1.6.scaffold1838.2 |
| CabHLH095 | Chr00 | 180717-183208 | 240 | 26.55 | 7.58 | 48.33 | CA.PGAv.1.6.scaffold1110.12 |
| CabHLH096 | Chr00 | 203429-207590 | 335 | 37.42 | 6.03 | 60.93 | CA.PGAv.1.6.scaffold588.25 |
| CabHLH097 | Chr00 | 267000-269813 | 471 | 53.02 | 6.34 | 50.50 | CA.PGAv.1.6.scaffold1412.16 |
| CabHLH098 | Chr00 | 326501-327401 | 243 | 27.16 | 8.44 | 56.26 | CA.PGAv.1.6.scaffold1030.14 |
| CabHLH099 | Chr00 | 329604-336894 | 722 | 77.55 | 7.27 | 56.82 | CA.PGAv.1.6.scaffold1248.10 |
| CabHLH100 | Chr00 | 486823-491334 | 224 | 24.92 | 6.31 | 71.97 | CA.PGAv.1.6.scaffold1030.24 |
| CabHLH101 | Chr00 | 533828-536897 | 320 | 35.39 | 6.41 | 32.92 | CA.PGAv.1.6.scaffold1041.44 |
| CabHLH102 | Chr00 | 645405-647964 | 263 | 30.18 | 7.08 | 51.94 | CA.PGAv.1.6.scaffold960.65 |
| CabHLH103 | Chr00 | 645450-649057 | 212 | 23.56 | 7.68 | 40.84 | CA.PGAv.1.6.scaffold681.51 |
| CabHLH104 | Chr00 | 671041-674189 | 322 | 35.73 | 6.33 | 30.02 | CA.PGAv.1.6.scaffold1231.38 |
| CabHLH105 | Chr00 | 807191-810009 | 322 | 35.51 | 6.33 | 25.45 | CA.PGAv.1.6.scaffold1041.46 |
| CabHLH106 | Chr00 | 978852-980345 | 337 | 37.22 | 5.07 | 71.84 | CA.PGAv.1.6.scaffold606.53 |
| CabHLH107 | Chr00 | 1093432-1094817 | 352 | 39.80 | 6.17 | 43.30 | CA.PGAv.1.6.scaffold504.64 |

Note: Sixteen *CabHLHs* (*CabHLH092-CabHLH107*) failed to located on any chromosomes. They were arranged on a putative-chromosome and the chromosome was designated as Chr00.

**Table S2.** **Functionally characterized partial bHLH proteins from tomato and *Arabidopsis thaliana***

| **Name** | **bHLH name** | **Subgroup** | **Gene functions** | **References** |
| --- | --- | --- | --- | --- |
| RGE1/  ZHOUPI | AtbHLH095 | IV | Regulates embryonic development  and endosperm breakdown | [1, 2] |
| ICE/  SCRM | AtbHLH116 | II | Control stomatal development; implicated in  the cold acclimation response and freezing  tolerance | [3] |
| ICE2/  SCRM2 | AtbHLH033 | II |  | [4] |
| bHLH04 | AtbHLH004 | II | Regulate glucosinolate biosynthesis by interacting MYB;  MYC2 also Respond to abscisic acid, jasmonic acid  and light signalling | [5] |
| bHLH05 | AtbHLH005 | II |  |  |
| MYC2 | AtbHLH006 | II |  | [6-8] |
| TT8 | AtbHLH042 | II | Partially redundantly regulate anthocyanin  biosynthesis, trichome and root hair development in combination with MYB and WD40 transcription factors | [9] |
| GL3 | AtbHLH001 | II |  | [10] |
| EGL3 | AtbHLH002 | II |  | [11] |
| PIF3 | AtbHLH008 | VI | Regulate anthocyanin biosynthesis by combining bZIP transcription factor | [12] |
| PIF4 | AtbHLH009 | VI | Bind to activated phytochromes and mediate  light and gibberellin signaling responses;  PIF4 also mediate plant architecture  response to high temperatures | [13, 14] |
| PIF5/  PIF6 | AtbHLH065 | VI |  | [15] |
| HFR1 | AtbHLH026 | VI | Mediate phytochrome and cryptochrome signaling mediator of germination responses to light and temperature | [16] |
| SPATULA | AtbHLH024 | VI |  | [17] |
| BEE1 | AtbHLH044 | VIII | Redundant positive regulators of brassinosteroid  signalling | [18] |
| BEE2 | AtbHLH058 | VIII |  |  |
| BEE3 | AtbHLH050 | VIII |  |  |
| SlICE1a | SlbHLH084 | II | Confer cold, osmotic and salt tolerance | [19] |
| SlAN1 | SlbHLH113 | II | Regulate anthocyanin biosynthesis | [20] |
| SlbHLH22 | SlbHLH046 | VIII | Promote early flowering and accelerate fruit ripening | [21] |
| SlPIF1a | SlbHLH111 | VI | Regulate carotenoid biosynthesis by a light-dependent mechanism | [22] |
| SlICE1 | SlbHLH050 | II | Involved in cold and salt stress signaling | [19] |
| SlMYC1 | SlbHLH098 | II | Regulate glandular trichome development  and terpene biosynthesis | [23] |
| SlMYC2 | SlbHLH104 | II | Regulate Steroidal glycoalkaloids biosynthesis | [24] |

**Table S3.** **Features of the CabHLH proteins motifs**

|  | **E-value** | **Sites** | **Width** | **Logo** |
| --- | --- | --- | --- | --- |
| 1 | 3.4e-1001 | 100 | 21 | 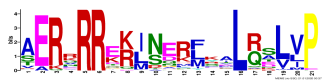 |
| 2 | 8.5e-1022 | 99 | 29 | 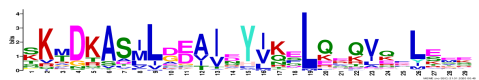 |
| 3 | 4.3e-254 | 35 | 37 | 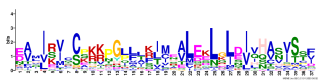 |
| 4 | 2.1e-237 | 5 | 100 | 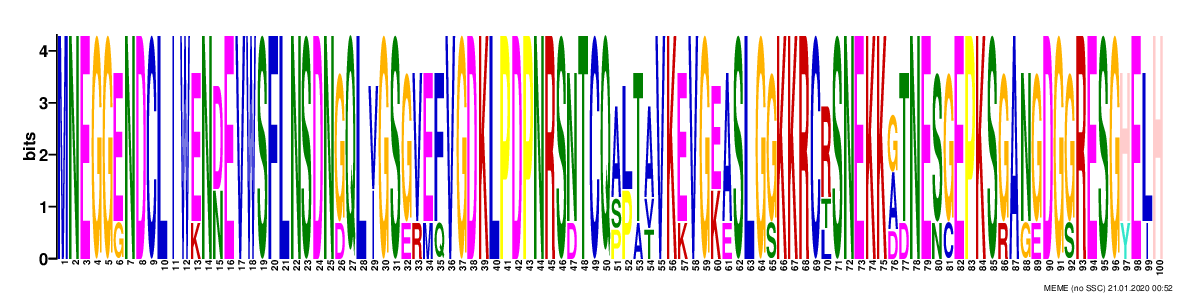 |
| 5 | 8.0e-181 | 22 | 15 | 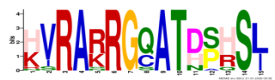 |
| 6 | 1.6e-096 | 13 | 33 | 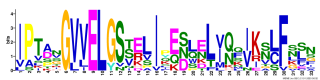 |
| 7 | 1.5e-103 | 8 | 37 | 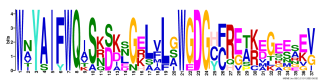 |
| 8 | 4.6e-078 | 5 | 62 | 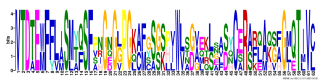 |
| 9 | 9.0e-073 | 5 | 30 | 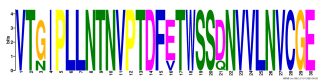 |
| 10 | 1.3e-067 | 6 | 69 | 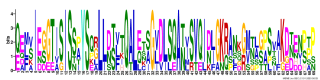 |
| 11 | 6.7e-059 | 7 | 29 | 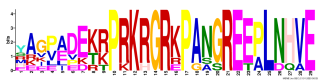 |
| 12 | 4.3e-055 | 5 | 29 | 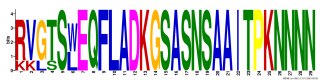 |
| 13 | 6.9e-052 | 5 | 30 | 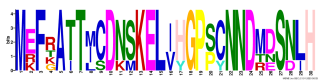 |
| 14 | 3.8e-039 | 6 | 47 | 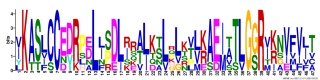 |
| 15 | 3.3e-033 | 5 | 21 | 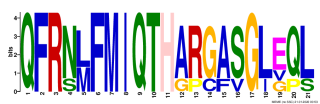 |

Note: The features of the CabHLH proteins motifs are predicted using MEME Suite 5.2.0.

**Table S4.** **List of the bHLH genes in rice, tomato and *Arabidopsis thaliana***

| **Rice** | | **Tomato** | | ***Arabidopsis thaliana*** | |
| --- | --- | --- | --- | --- | --- |
| **ID** | **Gene name** | **ID** | **Gene name** | **ID** | **Gene name** |
| BGIOSGA000097-PA | OsbHLH001 | Solyc00g050430.2 | SlbHLH001 | AT3G61950 | AtbHLH067 |
| BGIOSGA000206-PA | OsbHLH002 | Solyc01g010130.2 | SlbHLH002 | AT3G24140 | AtbHLH097 |
| BGIOSGA000262-PA | OsbHLH003 | Solyc01g014910.1 | SlbHLH003 | AT2G46810 | AtbHLH070 |
| BGIOSGA000686-PA | OsbHLH004 | Solyc01g020170.1 | SlbHLH004 | AT5G46690 | AtbHLH071 |
| BGIOSGA000939-PA | OsbHLH005 | Solyc01g057300.1 | SlbHLH005 | AT4G01460 | AtbHLH057 |
| BGIOSGA001324-PA | OsbHLH006 | Solyc01g067340.1 | SlbHLH006 | AT5G65320 | AtbHLH099 |
| BGIOSGA001389-PA | OsbHLH007 | Solyc01g080050.2 | SlbHLH007 | AT1G72210 | AtbHLH096 |
| BGIOSGA001523-PA | OsbHLH008 | Solyc01g081090.1 | SlbHLH008 | AT1G22490 | AtbHLH094 |
| BGIOSGA001526-PA | OsbHLH009 | Solyc01g081100.1 | SlbHLH009 | AT5G53210 | AtbHLH098 |
| BGIOSGA001870-PA | OsbHLH010 | Solyc01g086870.2 | SlbHLH010 | AT3G06120 | AtbHLH045 |
| BGIOSGA002533-PA | OsbHLH011 | Solyc01g090790.2 | SlbHLH011 | AT1G49770 | AtbHLH095 |
| BGIOSGA002600-PA | OsbHLH012 | Solyc01g096050.2 | SlbHLH012 | AT1G12540 | AtbHLH055 |
| BGIOSGA002940-PA | OsbHLH013 | Solyc01g096370.2 | SlbHLH013 | AT1G62975 | AtbHLH125 |
| BGIOSGA002941-PA | OsbHLH014 | Solyc01g098720.2 | SlbHLH014 | AT4G25410 | AtbHLH126 |
| BGIOSGA002943-PA | OsbHLH015 | Solyc01g102300.2 | SlbHLH015 | AT4G25400 | AtbHLH118 |
| BGIOSGA003013-PA | OsbHLH016 | Solyc01g106460.2 | SlbHLH016 | AT5G51790 | AtbHLH120 |
| BGIOSGA003814-PA | OsbHLH017 | Solyc01g107140.1 | SlbHLH017 | AT5G51780 | AtbHLH036 |
| BGIOSGA003819-PA | OsbHLH018 | Solyc01g107950.1 | SlbHLH018 | AT2G41240 | AtbHLH100 |
| BGIOSGA003821-PA | OsbHLH019 | Solyc01g107960.2 | SlbHLH019 | AT5G04150 | AtbHLH101 |
| BGIOSGA004292-PA | OsbHLH020 | Solyc01g107970.1 | SlbHLH020 | AT3G56970 | AtbHLH038 |
| BGIOSGA004736-PA | OsbHLH021 | Solyc01g109700.2 | SlbHLH021 | AT3G56980 | AtbHLH039 |
| BGIOSGA004859-PA | OsbHLH022 | Solyc01g111130.2 | SlbHLH022 | AT2G31210 | AtbHLH091 |
| BGIOSGA004992-PA | OsbHLH023 | Solyc02g062690.2 | SlbHLH023 | AT2G31220 | AtbHLH010 |
| BGIOSGA005178-PA | OsbHLH024 | Solyc02g063430.2 | SlbHLH024 | AT1G06170 | AtbHLH089 |
| BGIOSGA005408-PA | OsbHLH025 | Solyc02g070880.1 | SlbHLH025 | AT4G21330 | AtbHLH022 |
| BGIOSGA005582-PA | OsbHLH026 | Solyc02g076920.2 | SlbHLH026 | AT2G28160 | AtbHLH029 |
| BGIOSGA005697-PA | OsbHLH027 | Solyc02g078130.2 | SlbHLH027 | AT2G16910 | AtbHLH021 |
| BGIOSGA005771-PA | OsbHLH028 | Solyc02g079760.2 | SlbHLH028 | AT5G65640 | AtbHLH093 |
| BGIOSGA006236-PA | OsbHLH029 | Solyc02g079810.1 | SlbHLH029 | AT5G10570 | AtbHLH061 |
| BGIOSGA006748-PA | OsbHLH030 | Solyc02g079970.2 | SlbHLH030 | AT1G12860 | AtbHLH033 |
| BGIOSGA006856-PA | OsbHLH031 | Solyc02g084880.2 | SlbHLH031 | AT3G26744 | AtbHLH116 |
| BGIOSGA007229-PA | OsbHLH032 | Solyc02g090950.1 | SlbHLH032 | AT1G10610 | AtbHLH090 |
| BGIOSGA007247-PA | OsbHLH033 | Solyc02g091440.1 | SlbHLH033 | AT5G57150 | AtbHLH035 |
| BGIOSGA008112-PA | OsbHLH034 | Solyc02g091690.2 | SlbHLH034 | AT4G29930 | AtbHLH027 |
| BGIOSGA008398-PA | OsbHLH035 | Solyc02g091800.2 | SlbHLH035 | AT4G16430 | AtbHLH003 |
| BGIOSGA008576-PA | OsbHLH036 | Solyc02g091810.1 | SlbHLH036 | AT1G01260 | AtbHLH013 |
| BGIOSGA008846-PA | OsbHLH037 | Solyc02g091820.1 | SlbHLH037 | AT2G46510 | AtbHLH017 |
| BGIOSGA008903-PA | OsbHLH038 | Solyc02g093280.2 | SlbHLH038 | AT4G00870 | AtbHLH014 |
| BGIOSGA009202-PA | OsbHLH039 | Solyc03g005350.2 | SlbHLH039 | AT1G32640 | AtbHLH006 |
| BGIOSGA009622-PA | OsbHLH040 | Solyc03g006910.2 | SlbHLH040 | AT4G17880 | AtbHLH004 |
| BGIOSGA010080-PA | OsbHLH041 | Solyc03g007410.2 | SlbHLH041 | AT5G46760 | AtbHLH005 |
| BGIOSGA010239-PA | OsbHLH042 | Solyc03g031450.2 | SlbHLH042 | AT5G46830 | AtbHLH028 |
| BGIOSGA010315-PA | OsbHLH043 | Solyc03g034000.2 | SlbHLH043 | AT4G09820 | AtbHLH042 |
| BGIOSGA010604-PA | OsbHLH044 | Solyc03g044460.1 | SlbHLH044 | AT4G00480 | AtbHLH012 |
| BGIOSGA010986-PA | OsbHLH045 | Solyc03g095980.2 | SlbHLH045 | AT5G41315 | AtbHLH001 |
| BGIOSGA010988-PA | OsbHLH046 | Solyc03g097820.1 | SlbHLH046 | AT1G63650 | AtbHLH002 |
| BGIOSGA011172-PA | OsbHLH047 | Solyc03g113560.2 | SlbHLH047 | AT2G22770 | AtbHLH020 |
| BGIOSGA011179-PA | OsbHLH048 | Solyc03g114720.2 | SlbHLH048 | AT2G22760 | AtbHLH019 |
| BGIOSGA011251-PA | OsbHLH049 | Solyc03g115540.1 | SlbHLH049 | AT2G22750 | AtbHLH018 |
| BGIOSGA011499-PA | OsbHLH050 | Solyc03g118310.2 | SlbHLH050 | AT4G37850 | AtbHLH025 |
| BGIOSGA011741-PA | OsbHLH051 | Solyc03g119390.2 | SlbHLH051 | AT3G47640 | AtbHLH047 |
| BGIOSGA012015-PA | OsbHLH052 | Solyc03g120530.2 | SlbHLH052 | AT4G36060 | AtbHLH011 |
| BGIOSGA012750-PA | OsbHLH053 | Solyc03g121240.1 | SlbHLH053 | AT3G19860 | AtbHLH121 |
| BGIOSGA013197-PA | OsbHLH054 | Solyc04g005130.2 | SlbHLH054 | AT5G54680 | AtbHLH105 |
| BGIOSGA013293-PA | OsbHLH055 | Solyc04g005220.1 | SlbHLH055 | AT1G51070 | AtbHLH115 |
| BGIOSGA013493-PA | OsbHLH056 | Solyc04g005280.2 | SlbHLH056 | AT3G23210 | AtbHLH034 |
| BGIOSGA013533-PA | OsbHLH057 | Solyc04g006990.2 | SlbHLH057 | AT4G14410 | AtbHLH104 |
| BGIOSGA013600-PA | OsbHLH058 | Solyc04g007300.2 | SlbHLH058 | AT5G56960 | AtbHLH041 |
| BGIOSGA013618-PA | OsbHLH059 | Solyc04g007430.1 | SlbHLH059 | AT5G43650 | AtbHLH092 |
| BGIOSGA013672-PA | OsbHLH060 | Solyc04g014360.2 | SlbHLH060 | AT1G68810 | AtbHLH030 |
| BGIOSGA013729-PA | OsbHLH061 | Solyc04g074810.2 | SlbHLH061 | AT3G25710 | AtbHLH032 |
| BGIOSGA014424-PA | OsbHLH062 | Solyc04g076240.1 | SlbHLH062 | AT3G56770 | AtbHLH107 |
| BGIOSGA014565-PA | OsbHLH063 | Solyc04g077480.2 | SlbHLH063 | AT2G41130 | AtbHLH106 |
| BGIOSGA014566-PA | OsbHLH064 | Solyc04g078690.2 | SlbHLH064 | AT2G40200 | AtbHLH051 |
| BGIOSGA014567-PA | OsbHLH065 | Solyc04g078790.2 | SlbHLH065 | AT1G68240 | AtbHLH109 |
| BGIOSGA015290-PA | OsbHLH066 | Solyc04g080710.2 | SlbHLH066 | AT1G25310 | AtbHLH108 |
| BGIOSGA015389-PA | OsbHLH067 | Solyc05g005300.2 | SlbHLH067 | AT3G59060 | AtbHLH065 |
| BGIOSGA015472-PA | OsbHLH068 | Solyc05g006650.2 | SlbHLH068 | AT2G43010 | AtbHLH009 |
| BGIOSGA015604-PA | OsbHLH069 | Solyc05g009640.2 | SlbHLH069 | AT1G09530 | AtbHLH008 |
| BGIOSGA016251-PA | OsbHLH070 | Solyc05g009880.2 | SlbHLH070 | AT4G28800 | AtbHLH056 |
| BGIOSGA016427-PA | OsbHLH071 | Solyc05g010610.2 | SlbHLH071 | AT4G28815 | AtbHLH127 |
| BGIOSGA016651-PA | OsbHLH072 | Solyc05g011810.2 | SlbHLH072 | AT4G28811 | AtbHLH119 |
| BGIOSGA017025-PA | OsbHLH073 | Solyc05g014590.2 | SlbHLH073 | AT4G28790 | AtbHLH023 |
| BGIOSGA017139-PA | OsbHLH074 | Solyc05g050560.1 | SlbHLH074 | AT2G20180 | AtbHLH015 |
| BGIOSGA017200-PA | OsbHLH075 | Solyc05g053660.1 | SlbHLH075 | AT2G46970 | AtbHLH124 |
| BGIOSGA017471-PA | OsbHLH076 | Solyc06g008030.2 | SlbHLH076 | AT3G62090 | AtbHLH132 |
| BGIOSGA018982-PA | OsbHLH077 | Solyc06g035490.2 | SlbHLH077 | AT1G02340 | AtbHLH026 |
| BGIOSGA019157-PA | OsbHLH078 | Solyc06g051260.2 | SlbHLH078 | AT5G61270 | AtbHLH072 |
| BGIOSGA019218-PA | OsbHLH079 | Solyc06g051550.2 | SlbHLH079 | AT4G00050 | AtbHLH016 |
| BGIOSGA020126-PA | OsbHLH080 | Solyc06g062460.2 | SlbHLH080 | AT5G67110 | AtbHLH073 |
| BGIOSGA020128-PA | OsbHLH081 | Solyc06g064580.1 | SlbHLH081 | AT4G36930 | AtbHLH024 |
| BGIOSGA020285-PA | OsbHLH082 | Solyc06g064590.1 | SlbHLH082 | AT3G22100 | AtbHLH117 |
| BGIOSGA020491-PA | OsbHLH083 | Solyc06g065040.2 | SlbHLH083 | AT1G30670 | AtbHLH052 |
| BGIOSGA020891-PA | OsbHLH084 | Solyc06g068870.2 | SlbHLH084 | AT2G34820 | AtbHLH053 |
| BGIOSGA021743-PA | OsbHLH085 | Solyc06g069370.2 | SlbHLH085 | AT3G21330 | AtbHLH087 |
| BGIOSGA021777-PA | OsbHLH086 | Solyc06g069600.1 | SlbHLH086 | AT5G67060 | AtbHLH088 |
| BGIOSGA022375-PA | OsbHLH087 | Solyc06g072520.1 | SlbHLH087 | AT3G50330 | AtbHLH037 |
| BGIOSGA022533-PA | OsbHLH088 | Solyc06g083170.2 | SlbHLH088 | AT5G09750 | AtbHLH043 |
| BGIOSGA022687-PA | OsbHLH089 | Solyc06g083980.1 | SlbHLH089 | AT4G00120 | AtbHLH040 |
| BGIOSGA022967-PA | OsbHLH090 | Solyc07g005400.2 | SlbHLH090 | AT1G27740 | AtbHLH054 |
| BGIOSGA023024-PA | OsbHLH091 | Solyc07g018010.2 | SlbHLH091 | AT2G14760 | AtbHLH084 |
| BGIOSGA023114-PA | OsbHLH092 | Solyc07g039570.2 | SlbHLH092 | AT4G33880 | AtbHLH085 |
| BGIOSGA023624-PA | OsbHLH093 | Solyc07g043580.2 | SlbHLH093 | AT1G66470 | AtbHLH083 |
| BGIOSGA023869-PA | OsbHLH094 | Solyc07g052930.2 | SlbHLH094 | AT5G37800 | AtbHLH086 |
| BGIOSGA023988-PA | OsbHLH095 | Solyc07g053290.2 | SlbHLH095 | AT2G42280 | AtbHLH130 |
| BGIOSGA024146-PA | OsbHLH096 | Solyc07g063830.2 | SlbHLH096 | AT1G05805 | AtbHLH128 |
| BGIOSGA024674-PA | OsbHLH097 | Solyc07g064040.2 | SlbHLH097 | AT2G43140 | AtbHLH129 |
| BGIOSGA025137-PA | OsbHLH098 | Solyc08g005050.2 | SlbHLH098 | AT1G51140 | AtbHLH122 |
| BGIOSGA025266-PA | OsbHLH099 | Solyc08g008600.2 | SlbHLH099 | AT4G09180 | AtbHLH081 |
| BGIOSGA025912-PA | OsbHLH100 | Solyc08g062780.1 | SlbHLH100 | AT1G35460 | AtbHLH080 |
| BGIOSGA026551-PA | OsbHLH101 | Solyc08g075090.2 | SlbHLH101 | AT3G19500 | AtbHLH113 |
| BGIOSGA026577-PA | OsbHLH102 | Solyc08g075110.1 | SlbHLH102 | AT3G20640 | AtbHLH123 |
| BGIOSGA026775-PA | OsbHLH103 | Solyc08g076820.2 | SlbHLH103 | AT4G29100 | AtbHLH068 |
| BGIOSGA026779-PA | OsbHLH104 | Solyc08g076930.1 | SlbHLH104 | AT2G20095 | AtbHLH133 |
| BGIOSGA026828-PA | OsbHLH105 | Solyc08g081140.2 | SlbHLH105 | AT4G21340 | AtbHLH103 |
| BGIOSGA026937-PA | OsbHLH106 | Solyc08g083170.1 | SlbHLH106 | AT4G05170 | AtbHLH114 |
| BGIOSGA027551-PA | OsbHLH107 | Solyc09g005070.1 | SlbHLH107 | AT1G61660 | AtbHLH112 |
| BGIOSGA027691-PA | OsbHLH108 | Solyc09g018130.1 | SlbHLH108 | AT4G30980 | AtbHLH069 |
| BGIOSGA027780-PA | OsbHLH109 | Solyc09g018150.1 | SlbHLH109 | AT2G24260 | AtbHLH066 |
| BGIOSGA028855-PA | OsbHLH110 | Solyc09g057710.2 | SlbHLH110 | AT5G58010 | AtbHLH082 |
| BGIOSGA028884-PA | OsbHLH111 | Solyc09g063010.2 | SlbHLH111 | AT1G03040 | AtbHLH007 |
| BGIOSGA028888-PA | OsbHLH112 | Solyc09g064920.2 | SlbHLH112 | AT4G02590 | AtbHLH059 |
| BGIOSGA028963-PA | OsbHLH113 | Solyc09g065100.1 | SlbHLH113 | AT1G25330 | AtbHLH075 |
| BGIOSGA029027-PA | OsbHLH114 | Solyc09g083220.2 | SlbHLH114 | AT1G73830 | AtbHLH050 |
| BGIOSGA029426-PA | OsbHLH115 | Solyc09g083360.2 | SlbHLH115 | AT1G18400 | AtbHLH044 |
| BGIOSGA029460-PA | OsbHLH116 | Solyc09g089870.2 | SlbHLH116 | AT4G34530 | AtbHLH063 |
| BGIOSGA029497-PA | OsbHLH117 | Solyc09g091760.1 | SlbHLH117 | AT1G68920 | AtbHLH049 |
| BGIOSGA029759-PA | OsbHLH118 | Solyc09g097870.2 | SlbHLH118 | AT1G26260 | AtbHLH076 |
| BGIOSGA029777-PA | OsbHLH119 | Solyc09g098110.2 | SlbHLH119 | AT5G48560 | AtbHLH078 |
| BGIOSGA030896-PA | OsbHLH120 | Solyc10g006510.2 | SlbHLH120 | AT3G07340 | AtbHLH062 |
| BGIOSGA030928-PA | OsbHLH121 | Solyc10g006640.2 | SlbHLH121 | AT1G10120 | AtbHLH074 |
| BGIOSGA030936-PA | OsbHLH122 | Solyc10g008260.1 | SlbHLH122 | AT3G23690 | AtbHLH077 |
| BGIOSGA030954-PA | OsbHLH123 | Solyc10g008270.2 | SlbHLH123 | AT2G42300 | AtbHLH048 |
| BGIOSGA030958-PA | OsbHLH124 | Solyc10g009270.2 | SlbHLH124 | AT3G57800 | AtbHLH060 |
| BGIOSGA031008-PA | OsbHLH125 | Solyc10g009290.1 | SlbHLH125 | AT1G59640 | AtbHLH031 |
| BGIOSGA031426-PA | OsbHLH126 | Solyc10g018510.1 | SlbHLH126 | AT5G62610 | AtbHLH079 |
| BGIOSGA031474-PA | OsbHLH127 | Solyc10g049720.1 | SlbHLH127 | AT4G36540 | AtbHLH058 |
| BGIOSGA032415-PA | OsbHLH128 | Solyc10g049780.1 | SlbHLH128 | AT2G18300 | AtbHLH064 |
| BGIOSGA032814-PA | OsbHLH129 | Solyc10g078380.1 | SlbHLH129 |  |  |
| BGIOSGA033493-PA | OsbHLH130 | Solyc10g079050.1 | SlbHLH130 |  |  |
| BGIOSGA033976-PA | OsbHLH131 | Solyc10g079070.1 | SlbHLH131 |  |  |
| BGIOSGA035358-PA | OsbHLH132 | Solyc10g079650.1 | SlbHLH132 |  |  |
| BGIOSGA035596-PA | OsbHLH133 | Solyc10g079660.1 | SlbHLH133 |  |  |
| BGIOSGA035894-PA | OsbHLH134 | Solyc10g079680.1 | SlbHLH134 |  |  |
| BGIOSGA035917-PA | OsbHLH135 | Solyc11g005780.1 | SlbHLH135 |  |  |
| BGIOSGA037460-PA | OsbHLH136 | Solyc11g010340.1 | SlbHLH136 |  |  |
| BGIOSGA037731-PA | OsbHLH137 | Solyc11g056650.1 | SlbHLH137 |  |  |
| BGIOSGA037732-PA | OsbHLH138 | Solyc12g010170.1 | SlbHLH138 |  |  |
| BGIOSGA037733-PA | OsbHLH139 | Solyc12g036430.1 | SlbHLH139 |  |  |
| BGIOSGA037734-PA | OsbHLH140 | Solyc12g036470.1 | SlbHLH140 |  |  |
| BGIOSGA037772-PA | OsbHLH141 | Solyc12g087850.1 | SlbHLH141 |  |  |
| BGIOSGA037775-PA | OsbHLH142 | Solyc12g088130.1 | SlbHLH142 |  |  |
| BGIOSGA037855-PA | OsbHLH143 | Solyc12g088790.1 | SlbHLH143 |  |  |
| BGIOSGA040642-PA | OsbHLH144 | Solyc12g098620.1 | SlbHLH144 |  |  |
|  |  | Solyc12g100140.1 | SlbHLH145 |  |  |

**Table S5.** **List of the primer for real-time quantitative PCR**

|  | **Primer name** | **Sequence** | **Melting temperatures (℃)** | **Annealing temperatures (℃)** | **The expected product sizes (bp)** |
| --- | --- | --- | --- | --- | --- |
| CabHLH007 | CabHLH007-F | CCACTGGTTCCACTCGTTCA | 80 | 57.4 | 226 |
|  | CabHLH007-R | CCTTTTTGTCTCTTCTTGCC |  | 53.4 |  |
| CabHLH009 | CaBHLH009-F | ATTTATGAGAGAAGTTGGGA | 80 | 49.2 | 122 |
|  | CaBHLH009-R | AGTTTGAGCTAGATGATTGG |  | 51.3 |  |
| CabHLH026 | CabHLH026-F | AGAAGATTCAGTTAGTTGCT | 79 | 49.2 | 158 |
|  | CabHLH026-R | TTAGATTTTTAGACGGTTGC |  | 49.2 |  |
| CabHLH063 | CabHLH063-F | ACACAGTCAAACACATAAAA | 81 | 47.2 | 190 |
|  | CabHLH063-R | TAAGAATGGAGAACAAAATA |  | 45.2 |  |
| CabHLH086 | CabHLH086-F | GTCCACTGGTTCCACTCCTT | 80 | 53.0 | 230 |
|  | CabHLH086-R | GTCCTTTTTGTCTCTTCTTA |  | 49.2 |  |
| CabHLH009 | CabHLH009-F | CTTGTTTTCCGTTGGTGGTG | 79 | 55.4 | 118 |
|  | CabHLH009-R | ATACGCTGACTGTTGTTGCT |  | 53.4 |  |
| CabHLH032 | CaBHLH032-F | ATTCTCAAAACCCTCACCCT | 83 | 56.0 | 228 |
|  | CaBHLH032-R | ACCACCTCCAGCACTACCAC |  | 61.5 |  |
| CabHLH048 | CaBHLH048-F | ATGTGGAGGCGGAGAGGCAG | 83 | 61.6 | 130 |
|  | CaBHLH048-R | TCAGTGATGTGAGCAATGGC |  | 55.4 |  |
| CabHLH095 | CaBHLH095-F | TTGTTACTTCCTTTCTGTTG | 80 | 49.2 | 250 |
|  | CaBHLH095-R | GTCCTGTGAGGTATCTATTG |  | 53.4 |  |
| CabHLH100 | CaBHLH100-F | ATATGTTCCTGGGTCTTTGC | 81 | 53.4 | 174 |
|  | CaBHLH100-R | GAGGTTATTGTTGGTTTGCC |  | 53.4 |  |
| AT3 | AT3-F | AAACCTTCCTCTCTCACCCCCTCT | 79 | 61.0 | 234 |
|  | AT3-R | AGCATTGTCCTTCAACTTTCCAGC |  | 58.0 |  |
| AMT | AMT-F | TCCTAAATATGTTTACTGCAAA | 83 | 51.0 | 283 |
|  | AMT-R | TCACCTGGCAAGTGATAG |  | 53.0 |  |
| BCKDH | BCKDH-F | TGCTTCTGCTGCTGCTCTATCTC | 80 | 62.0 | 141 |
|  | BCKDH-R | CTGCCTTTCCCTTTATCATCCTT |  | 58.0 |  |
| KasIa | KasIa-F | TTATAGGTTCTTTCTCCCCA | 83 | 51.0 | 250 |
|  | KasIa-R | GCCATTACATTCTTATGTTTGTC |  | 52.0 |  |
| Acl | Acl-F | TGGCTTCTATTACTGCATCTTC | 83 | 54.0 | 178 |
|  | Acl-R | CACACTTTGTCAACTGTCTCTG |  | 56.0 |  |
| CA00g52140 | CA00g52140-F | GGTCGCTTGGTTATGGTTAT | 81 | 53.4 | 130 |
|  | CA00g52140-R | ACAGTAGGGTCTCGGTTTGA |  | 55.4 |  |
| CA12g20490 | CA12g20490-F | GAAGACCCTGACGGGCAAGAC | 85 | 61.5 | 219 |
|  | CA12g20490-R | TTAGCACCACCACGGAGACGA |  | 59.5 |  |

**Table S6.** **List of the primer for yeast two-hybrid assays**

|  | **Primer name** | **Sequence** | **Annealing temperatures (**℃) | **The expected product sizes (bp)** |
| --- | --- | --- | --- | --- |
| MYB31 | AD-MYB31-F | gccatggaggccagtgaattcATGGTGAGAACACCTTGCTACGA | 61.4 | 750 |
|  | AD-MYB31-R | cagctcgagctcgatggatccTTACCAATAATTATAATGATCAAAGTCAAAG | 60.8 |  |
|  | BD-MYB31-F | atggccatggaggccgaattcATGGTGAGAACACCTTGCTACGA | 61.4 |  |
|  | BD-MYB31-R | ccgctgcaggtcgacggatccTTACCAATAATTATAATGATCAAAGTCAAAG | 60.8 |  |
| CabHLH007 | AD-CabHLH007-F | gccatggaggccagtgaattcATGGAAAGAGCATTGGAATGGT | 60.3 | 1443 |
|  | AD-CabHLH007-R | cagctcgagctcgatggatccTCAACTTGTCAACTCCATTAGTGAGG | 61.9 |  |
|  | BD-CabHLH007-F | atggccatggaggccgaattcATGGAAAGAGCATTGGAATGGT | 60.3 |  |
|  | BD-CabHLH007-R | ccgctgcaggtcgacggatccTCAACTTGTCAACTCCATTAGTGAGG | 61.9 |  |
| CabHLH009 | AD-CabHLH009-F | gccatggaggccagtgaattcATGTTTAGTTCAGAGCCAATTTCG | 60.1 | 1185 |
|  | AD-CabHLH009-R | cagctcgagctcgatggatccTCATGTTGAATATTCCAGCTGCTT | 61.0 |  |
|  | BD-CabHLH009-F | atggccatggaggccgaattcATGTTTAGTTCAGAGCCAATTTCG | 60.1 |  |
|  | BD-CabHLH009-R | ccgctgcaggtcgacggatccTCATGTTGAATATTCCAGCTGCTT | 61.0 |  |
| CaBHLH026 | AD-CabHLH026-F | gccatggaggccagtgaattcATGGAGCTTACTCAACAGGATTTTC | 60.5 | 1029 |
|  | AD-CabHLH026-R | cagctcgagctcgatggatccCTACAGACATCTTCCTCCATAACCTG | 60.8 |  |
|  | BD-CabHLH026-F | atggccatggaggccgaattcATGGAGCTTACTCAACAGGATTTTC | 60.5 |  |
|  | BD-CabHLH026-R | ccgctgcaggtcgacggatccCTACAGACATCTTCCTCCATAACCTG | 60.8 |  |
| CaBHLH063 | AD-CabHLH063-F | gccatggaggccagtgaattcATGCCCCCTCCAAGATTTAACA | 62.5 | 1089 |
|  | AD-CabHLH063-R | cagctcgagctcgatggatccTCAAGTGGTGGGTGCATTACTG | 60.8 |  |
|  | BD-CabHLH063-F | atggccatggaggccgaattcATGCCCCCTCCAAGATTTAACA | 62.5 |  |
|  | BD-CabHLH063-R | ccgctgcaggtcgacggatccTCAAGTGGTGGGTGCATTACTG | 60.8 |  |
| CabHLH086 | AD-CabHLH086-F | gccatggaggccagtgaattcATGGAAAGAGCATTGGAATGTTTA | 60.2 | 1443 |
|  | AD-CabHLH086-R | cagctcgagctcgatggatccTTAACTTGTCAACTCAATTAGTGAGGATC | 61.5 |  |
|  | BD-CabHLH086-F | atggccatggaggccgaattcATGGAAAGAGCATTGGAATGTTTA | 60.2 |  |
|  | BD-CabHLH086-R | ccgctgcaggtcgacggatccTTAACTTGTCAACTCAATTAGTGAGGATC | 61.5 |  |

Note: AD and BD represent empty pGADT7 and pGBKT7 vectors, separately.

**Table S7.** Putative *cis*-elements of [capsorubin](javascript:;) and capsaicin biosynthetic genes promoters

| Gene name | Promoter *cis*-element | The numbers of the bHLH DNA binding site |
| --- | --- | --- |
| *CCS* | AT1-motif (1) AT~TATA-box (3) CAAT-box (48) CAT-box (1) GATA-motif (1) myb (1) STRE (2) TATA-box (28) TCA-element (1) | 0 |
| *PSY* | 3-AF3 binding site (1) ABRE (1) ABRE3a (1) ABRE4 (1) AC-I (2) ARE (2) BOX II (1) BOX II-like sequence (1) CAAT-box (21) CAT-box (1) G-box (1) MBS (1) MYB (3) MYB-like sequence (1) MYC (2) Myb (1) STRE (2) Sp1 (4) TATA (1) TATA-box (27) TCT-motif (1) W box (1) | 2 |
| *β-CH* | ABRE (2) AE-box (1) ARE (2) AT~TATA-box (3) Box 4 (5) Box II (1) CAAT-box (33) CAT-box (1) ERE (1) G-box (1) STRE (2) TATA-box (47) TGA-element (1) WRE3 (1) | 0 |
| *β-LCY* | AAGAA-motif (1) ARE (4) AT~TATA-box (2) Box 4 (2) CAAT-box (41) ERE (1) GT1-motif (2) LTR (1) MRE (1) MYB (3) MYB-like sequence (2) MYC (6) Myc (1) STRE (2) TATA-box (42) TCA-element (1) W box (1) chs-CMA1a (1) | 6 |
| *Acl* | ABRE (1) AE-box (2) ARE (6) AT-rich element (2) AT1-motif (1) Box 4 (2) Box III (1) CAAT-box (39) CAT-box (1) CGTCA-motif (1) ERE (3) G-Box (1) GT1-motif (1) MYB (1) MYB-like sequence (1) MYC (2) STRE (1) TATA (1) TATA-box (19) TGACG-motif (1)  W box (2) WUN-motif (2) as-1 (1) | 2 |
| *AMT* | AAGAA-motif (1) ABRE (5) AT~TATA-box (5) Box 4 (3) CAAT-box (47) ERE (1) G-Box (2) G-box (3) GT1-motif (2) MBS (1) MRE (1) MYB (3) MYB-like sequence (2) MYC (5) Myb (1) Myb-binding site (1) O2-site (1) STRE (2) TATA-box (59) TCA (1) TCT-motif (1) W box (1) WUN-motif (3) chs-CMA2a (1) | 8 |
| *AT3* | ABRE (2) ARE (1) AT-rich element (1) AT~TATA-box (4) Box 4 (4) CAAT-box (31) DRE core (1) ERE (2) G-Box (1) G-box (1)  GT1-motif (1) Gap-box (1) MBSI (1) MYC (3) P-box (1) STRE (1) TATA-box (40) TCT-motif (1) | 4 |
| *BACT* | ABRE (6) ABRE3a (1) ABRE4 (1) ACE (1) ARE (2) AT-rich element (1) ATC-motif (1) AT~TATA-box (5) Box 4 (2) CAAT-box (27) CGTCA-motif (2) ERE (2) G-Box (3) G-box (2) GARE-motif (1) GATA-motif (1) GT1-motif (1) LTR (1) MYB (2) MYB-like sequence (1) MYC (2) Myb-binding site (1) Myc (1) TATA-box (47) TATC-box (1) TCA-element (2) TCT-motif (1) TGA-element (1) TGACG-motif (2) WUN-motif (2) as-1 (2) | 4 |
| *BCKDH* | A-box (1) AAGAA-motif (1) ABRE (1) ABRE3a (1) ABRE4 (1) AT-rich sequence (2) AT~TATA-box (3) Box 4 (5) Box II (1)  CAAT-box (26) CCGTCC motif (1) CCGTCC-box (1) ERE (6) G-box (1) GT1-motif (4) LAMP-element (1) MBSI (1) MYB (1)  MYB-like sequence (1) MYC (2) Myc (1) O2-site (2) TATA (1) TATA-box (38) TCT-motif (1) box S (1) | 3 |
| *CoMTa* | AAGAA-motif (1) ARE (1) AT-rich element (1) AT~TATA-box (6) AuxRR-core (1) Box 4 (1) CAAT-box (24) CGTCA-motif (1)  ERE (4) GATA-motif (1) GT1-motif (1) I-box (3) MYB (1) MYB-like sequence (1) MYC (2) Myb (1) Myc (1) STRE (1) TATA (1)  TATA-box (34) TCT-motif (1) TGACG-motif (1) WUN-motif (2) as-1 (1) | 2 |
| *FatA* | ARE (1) AT1-motif (1) At~TATA-box (4) Box 4 (4) CAAT-box (32) CAT-box (1) CGTCA-motif (1) ERE (1) GCN4_motif (1) LTR (1)  MBS (1) MYC (3) Myb (2) Myc (1) P-box (1) STRE (3) TATA (1) TATA-box (58) TCA-element (1) TGACG-motif (1) as-1 (1) | 3 |
| *KasIa* | AE-box (1) ARE (3) AT~TATA-box (2) Box 4 (1) CAAT-box (17) CGTCA-motif (1) GATA-motif (1) GC-motif (1) I-box (1) LTR (2)  P-box (1) STRE (1) TATA-box (25) TCA-element (1) TGACG-motif (1) WRE3 (1) as-1 (1) circadian (1) re2f-1 (1) | 0 |

Note: 1500bp nucleotide sequences upstream of the start codon (ATG) from [capsorubin](javascript:;) and capsaicin biosynthetic genes including *CCS*, *PSY*, *β-CH*, *β-LCY*, *Acl*, *AMT*, *AT3*, *BACT,* *BCKDH*, *CoMTa*, *FatA* and *KasIa* were extracted from *Capsicum annuum* genome. The *cis*-elements were found using PlantCARE database (<http://bioinformatics.psb.ugent.be/webtools/plantcare/html/>). Arabic numbers in the parentheses indicated the number of corresponding *cis*-element. The basis region of bHLH protein could bind to consensus hexanucleotide E-box (CANNTG). The G-box (CACGTG) and the MYC (CATTTG) are the common forms of E-box.

**References**

1. Kondou Y, Nakazawa M, Kawashima M, Ichikawa T, Yoshizumi T, Suzuki K, Ishikawa A, Koshi T, Matsui R, Muto S. RETARDED GROWTH OF EMBRYO1, a new basic helix-loop-helix protein, expresses in endosperm to control embryo growth. Plant Physiol. 2008;147(4):1924-1935.

2. Yang S, Johnston N, Talideh E, Mitchell S, Jeffree C, Goodrich J, Ingram G. The endosperm-specific *ZHOUPI* gene of *Arabidopsis thaliana* regulates endosperm breakdown and embryonic epidermal development. Development. 2008;135(21):3501-3509.

3. Chinnusamy V, Ohta M, Kanrar S, Lee BH, Hong X, Agarwal M, Zhu JK. ICE1: a regulator of cold-induced transcriptome and freezing tolerance in *Arabidopsis*. Genes Dev. 2003;17(8):1043-1054.

4. Fursova OV, Pogorelko GV, Tarasov VA. Identification of *ICE2*, a gene involved in cold acclimation which determines freezing tolerance in *Arabidopsis thaliana*. Gene. 2009;429(1-2):98-103.

5. Frerigmann H, Berger B, Gigolashvili T. bHLH05 is an interaction partner of MYB51 and a novel regulator of glucosinolate biosynthesis in *Arabidopsis*. Plant Physiol. 2014;166(1):349-369.

6. Abe H, Urao T, Ito T, Seki M, Shinozaki K, Yamaguchi-Shinozaki K. *Arabidopsis* AtMYC2 (bHLH) and AtMYB2 (MYB) function as transcriptional activators in abscisic acid signaling. Plant Cell. 2003;15(1):63-78.

7. Lorenzo O, Chico JM, Sanchez-Serrano JJ, Solano R. JASMONATE-INSENSITIVE1 encodes a MYC transcription factor essential to discriminate between different jasmonate-regulated defense responses in *Arabidopsis*. Plant Cell. 2004;16(7):1938-1950.

8. Yadav V, Mallappa C, Gangappa SN, Bhatia S, Chattopadhyay S. A basic helix-loop-helix transcription factor in *Arabidopsis*, MYC2, acts as a repressor of blue light-mediated photomorphogenic growth. Plant Cell. 2005;17(7):1953-1966.

9. Nesi N, Debeaujon I, Jond C, Pelletier G, Caboche M, Lepiniec L. The *TT8* gene encodes a basic helix-loop-helix domain protein required for expression of *DFR* and *BAN* genes in *Arabidopsis* siliques. Plant Cell. 2000;12(10):1863-1878.

10. Payne CT, Zhang F, Lloyd AM. *GL3* encodes a bHLH protein that regulates trichome development in *Arabidopsis* through interaction with GL1 and TTG1. Genetics. 2000;156(3):1349-1362.

11. Bernhardt C, Lee MM, Gonzalez A, Zhang F, Lloyd A, Schiefelbein J. The bHLH genes *GLABRA3 (GL3)* and *ENHANCER OF GLABRA3 (EGL3)* specify epidermal cell fate in the *Arabidopsis* root. Development. 2003;130(26):6431-6439.

12. Shin J, Park E, Choi G. PIF3 regulates anthocyanin biosynthesis in an HY5-dependent manner with both factors directly binding anthocyanin biosynthetic gene promoters in *Arabidopsis*. Plant J. 2007;49(6):981-994.

13. Leivar P, Monte E, Al-Sady B, Carle C, Storer A, Alonso JM, Ecker JR, Quail PH. The *Arabidopsis* phytochrome-interacting factor PIF7, together with PIF3 and PIF4, regulates responses to prolonged red light by modulating phyB levels. Plant Cell. 2008;20(2):337-352.

14. Koini MA, Alvey L, Allen T, Tilley CA, Harberd NP, Whitelam GC, Franklin KA. High temperature-mediated adaptations in plant architecture require the bHLH transcription factor PIF4. Curr Biol. 2009;19(5):408-413.

15. Castillon A, Shen H, Huq E. Phytochrome interacting factors: central players in phytochrome-mediated light signaling networks. Trends Plant Sci. 2007;12(11):514-521.

16. Duek PD, Fankhauser C. HFR1, a putative bHLH transcription factor, mediates both phytochrome A and cryptochrome signalling. Plant J. 2003;34(6):827-836.

17. Penfield S, Josse EM, Kannangara R, Gilday AD, Halliday KJ, Graham IA. Cold and light control seed germination through the bHLH transcription factor SPATULA. Curr Biol. 2005;15(22):1998-2006.

18. Friedrichsen DM, Nemhauser J, Muramitsu T, Maloof JN, Alonso J, Ecker JR, Furuya M, Chory J. Three redundant brassinosteroid early response genes encode putative bHLH transcription factors required for normal growth. Genetics. 2002;162(3):1445-1456.

19. Feng HL, Ma NN, Meng X, Zhang S, Wang JR, Chai S, Meng QW. A novel tomato MYC-type ICE1-like transcription factor, SlICE1a, confers cold, osmotic and salt tolerance in transgenic tobacco. Plant Physiol Biochem. 2013;73:309-320.

20. Park J, Nou I, Kim H, Afrin KS, Kang S, Rahim MA, Jung H. Expression of anthocyanin biosynthesis-related genes reflects the peel color in purple tomato. Hortic Environ Biote. 2018;59(3):435-445.

21. Waseem M, Li N, Su D, Chen J, Li Z. Overexpression of a basic helix-loop-helix transcription factor gene, *SlbHLH22*, promotes early flowering and accelerates fruit ripening in tomato (*Solanum lycopersicum L.*). Planta. 2019;250(1):173-185.

22. Llorente B, D'Andrea L, Ruiz-Sola MA, Botterweg E, Pulido P, Andilla J, Loza-Alvarez P, Rodriguez-Concepcion M. Tomato fruit carotenoid biosynthesis is adjusted to actual ripening progression by a light-dependent mechanism. Plant J. 2016;85(1):107-119.

23. Xu J, van Herwijnen ZO, Drager DB, Sui C, Haring MA, Schuurink RC. SlMYC1 regulates type VI glandular trichome formation and terpene biosynthesis in tomato glandular cells. Plant Cell. 2018;30(12):2988-3005.

24. Cardenas PD, Sonawane PD, Pollier J, Vanden BR, Dewangan V, Weithorn E, Tal L, Meir S, Rogachev I, Malitsky S. GAME9 regulates the biosynthesis of steroidal alkaloids and upstream isoprenoids in the plant mevalonate pathway. Nat Commun. 2016;7(10654):10654.
